# Characterization of the total and viable bacterial and fungal communities associated with the International Space Station surfaces

**DOI:** 10.1186/s40168-019-0666-x

**Published:** 2019-04-08

**Authors:** Aleksandra Checinska Sielaff, Camilla Urbaniak, Ganesh Babu Malli Mohan, Victor G. Stepanov, Quyen Tran, Jason M. Wood, Jeremiah Minich, Daniel McDonald, Teresa Mayer, Rob Knight, Fathi Karouia, George E. Fox, Kasthuri Venkateswaran

**Affiliations:** 1 Jet Propulsion Laboratory, California Institute of Technology, Biotechnology and Planetary Protection Group,, Pasadena, CA USA; 2 Department of Biology and Biochemistry, University of Houston, Houston, TX USA; 3 Marine Biology Research Division, Scripps Institute of Oceanography, University of California San Diego, La Jolla, CA USA; 4 Department of Pediatrics, University of California San Diego, La Jolla, CA USA; 5 Center for Microbiome Innovation, University of California San Diego, La Jolla, CA USA; 6 Department of Computer Science and Engineering, University of California San Diego, La Jolla, CA USA; 7 NASA Ames Research Center, Space Bioscience Division, Moffett Field, Mountain View, CA USA; 8 Research Center, Moffett Field, Mountain View, CA USA; 9 Department of Pharmaceutical Chemistry, University of California San Francisco, San Francisco, CA USA; 10 Washington State University Extension – Youth and Families Program Unit, Washington State University, Pullman, WA USA

**Keywords:** International Space Station, Microbiome, 16S rRNA, ITS, Environmental surface, Built microbiome, Propidium monoazide, Microbial diversity

## Abstract

**Background:**

The International Space Station (ISS) is a closed system inhabited by microorganisms originating from life support systems, cargo, and crew that are exposed to unique selective pressures such as microgravity. To date, mandatory microbial monitoring and observational studies of spacecraft and space stations have been conducted by traditional culture methods, although it is known that many microbes cannot be cultured with standard techniques. To fully appreciate the true number and diversity of microbes that survive in the ISS, molecular and culture-based methods were used to assess microbial communities on ISS surfaces. Samples were taken at eight pre-defined locations during three flight missions spanning 14 months and analyzed upon return to Earth.

**Results:**

The cultivable bacterial and fungal population ranged from 10^4^ to 10^9^ CFU/m^2^ depending on location and consisted of various bacterial (*Actinobacteria*, *Firmicutes*, and *Proteobacteria*) and fungal (*Ascomycota* and *Basidiomycota*) phyla. Amplicon sequencing detected more bacterial phyla when compared to the culture-based analyses, but both methods identified similar numbers of fungal phyla. Changes in bacterial and fungal load (by culture and qPCR) were observed over time but not across locations. Bacterial community composition changed over time, but not across locations, while fungal community remained the same between samplings and locations. There were no significant differences in community composition and richness after propidium monoazide sample treatment, suggesting that the analyzed DNA was extracted from intact/viable organisms. Moreover, approximately 46% of intact/viable bacteria and 40% of intact/viable fungi could be cultured.

**Conclusions:**

The results reveal a diverse population of bacteria and fungi on ISS environmental surfaces that changed over time but remained similar between locations. The dominant organisms are associated with the human microbiome and may include opportunistic pathogens. This study provides the first comprehensive catalog of both total and intact/viable bacteria and fungi found on surfaces in closed space systems and can be used to help develop safety measures that meet NASA requirements for deep space human habitation. The results of this study can have significant impact on our understanding of other confined built environments on the Earth such as clean rooms used in the pharmaceutical and medical industries.

**Electronic supplementary material:**

The online version of this article (10.1186/s40168-019-0666-x) contains supplementary material, which is available to authorized users.

## Introduction

The International Space Station (ISS) is the largest human space platform in low Earth orbit (~ 400 km above Earth’s surface) and for the last 17 years it has been continuously inhabited by an international community of astronauts performing space research. The ISS is a hermetically sealed closed system, subjected to microgravity, radiation, elevated carbon dioxide, and the recirculation of air through HEPA filters and is considered an “extreme environment” [1, 2]. Microbes are known to survive and even thrive in extreme environments, and the microbes that are present on the ISS may have existed since the inception of the ISS while others may be introduced each time new astronauts or payloads arrive.

Since the beginning of the ISS, routine microbial monitoring of surfaces, air, and water has occurred using culture-based techniques as per the National Aeronautics and Space Administration’s (NASA) operations and maintenance requirement procedures [3]. However, culture-based analysis limits our understanding of the diversity of microbes that grow and thrive on the ISS because only a small fraction of organisms in a given environment can be cultured under standard laboratory conditions [4]. Molecular methods, such as quantitative polymerase chain reaction (qPCR) and targeted amplicon sequencing, which can identify and quantify both culturable and unculturable organisms provide a more thorough assessment of what is actually present and in what amounts [5]. However, while it has been recently shown as a proof of concept that PCR [6] and amplicon sequencing can be performed on the ISS [7, 8], microbial monitoring of the ISS with molecular-based methods is not routinely used because of the lack of simple, compact, and reliable sample processing instruments onboard the ISS. Once such devices are available, rapid, real-time microbial detection, functional analysis are possible for the long duration missions, but baseline information about the ISS environmental microbiome is still needed.

The importance of cataloging the ISS microbiome, which consists of both culturable and unculturable microbes, parallels the surge in research into the “built microbiome” here on Earth. Emerging studies on the microbiome of homes [9–11], offices, classrooms, museums [12, 13], and hospitals [5, 14, 15] have revealed an assemblage of bacteria, fungi, viruses, and protozoa unique to that indoor environment that are influenced by a variety of factors such as building design, ventilation, humidity, air pressure and flow, occupant numbers, or activities performed [16, 17]. Specific microbes in these indoor spaces have been shown to impact human health by influencing our susceptibility to allergies, infectious diseases, or sick building syndrome [18]. The influence of the indoor microbiome on human health becomes more important for astronauts during flights due to altered immunity associated with space flight [19, 20] and the lack of sophisticated medical interventions that are available on Earth.

In light of an upcoming new era of human expansion in the universe, such as future space travel to Mars, the microbiome of the closed space environment needs to be examined thoroughly to identify the types of microorganisms that can accumulate in this unique environment, how long they persist and survive, and their impact on human health and spacecraft infrastructure. For this reason, the National Research Council (NRC) Decadal Survey recommended that NASA establish a coordinated, large-scale Microbial Observatory program within the ISS platform [21]. As part of this NASA initiative, the microbial communities on ISS surfaces from eight defined locations over three flight missions, spanning 14 months, were characterized using culture-based techniques, qPCR, and amplicon sequencing of the 16S rRNA gene and internal transcribed spacer (ITS) region. Before DNA extraction, half of the sample was treated with propidium monoazide (PMA) so that the microbiome of intact/viable cells (PMA treatment) could be characterized. The PMA-untreated samples yielded information about the total microbial population (including free DNA, dead cells, cells with a compromised cell membrane, intact cells, and viable cells). PMA binds to DNA, making the DNA unavailable for amplification during PCR steps [22]. Due to its higher molecular weight and/or charge, PMA cannot penetrate into cells that have an intact cell membrane (i.e., viable) but can bind to free floating DNA or DNA inside cells with a compromised cell membrane (i.e., dead cells) [22, 23]. It is in this way that many studies have utilized PMA to distinguish between intact/viable cells and compromised/dead cells [2, 24–26].

This comprehensive analysis of the ISS microbiome was used to assess how microbial communities change over time (temporal distribution) and throughout the ISS (spatial distribution). In addition, the ISS environmental microbiome data were compared with other Earth built environmental microbiome data such as the Earth Microbiome Project [27], Hospital ([28], Qiita study 10,172), and Office microbiome ([28], Qiita study 10,423). The implementation of novel molecular techniques to monitor intact microbial populations in this unique environment opens a possibility for broadening the current surveillance practices to maintain the health of the crew and to promote advances in deep space human habitation in the future.

## Results

**Fig. 1:**
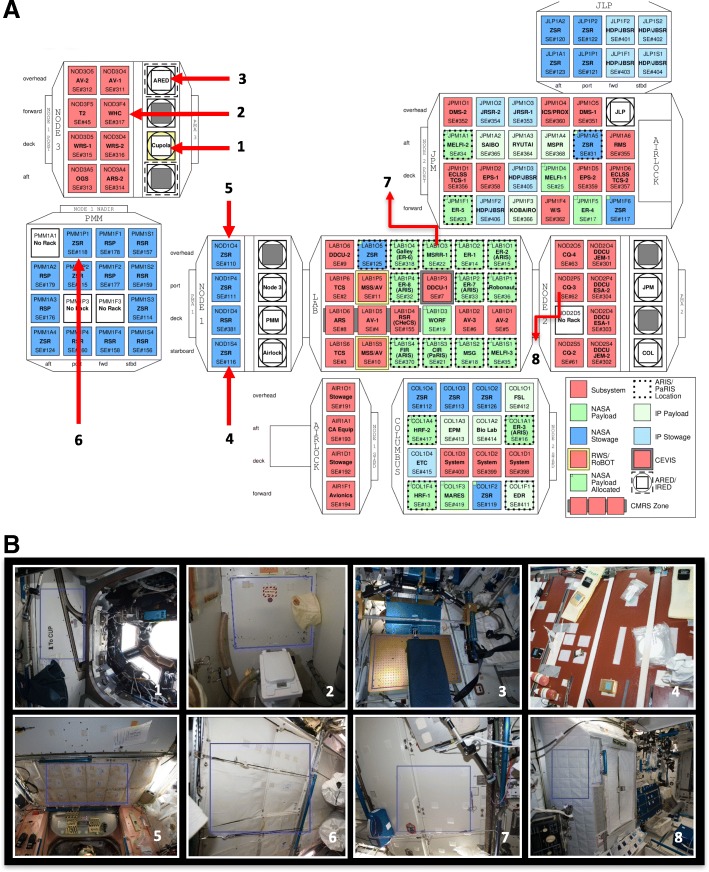
Illustration of the eight locations sampled on the ISS over three flight sampling sessions. **a** Schematic of the US module of the ISS depicting various nodes and modules. The red arrows point to locations sampled during this study. **b** Detailed images of the sampled area at each location as outlined by blue lines. Location #1, port panel next to cupola (Node 3); location #2, waste and hygiene compartment (node 3); location #3, advanced resistive exercise device (ARED) foot platform (node 3); location #4, dining table (node 1); location #5, zero G stowage rack (node 1); location #6, permanent multipurpose module (PMM) port 1 (PMM); location #7, panel near portable water dispenser (LAB); and location #8, port crew quarters, bump out exterior aft wall (node 2)

Twenty-four surface wipes were collected from eight locations across the ISS during three flight missions over the course of 14 months. In addition to these 24 wipes, wipes that were taken out of the kits and exposed to the ISS environment, but not used for sampling, were designated as controls and processed in parallel with the sample wipes. A summary of the sampled locations and the associated metadata is presented in Fig. 1 and Table 1.

**Table 1:** Description of ISS locations and associated metadata, from which surface swabs were collected

Location number	Location description	ISS module	
1	Port panel next to cupola	Node 3	
2	Waste and hygiene compartment	Node 3 “F4”	
3	Advanced resistive exercise device (ARED) foot platform	Node 3	
4	Dining table	Node 1	
5	Overhead 4	Node 1	
6	Permanent multipurpose module (PMM) Port 1	PMM	
7	Lab 3 overhead	LAB	
8	Port crew quarters, bump out exterior aft wall	Node 2	
Environmental parameters	Flight 1 (F1)	Flight 2 (F2)	Flight3 (F3)
Sampling date	March 4th 2015	May 15th 2015	May 6th 2016
Vehicle (ascent/descent)	SpX-5/TMA-14A	SpX-6/SpX-6	SpX-8/SpX-8
Crewmember who performed sampling	T. Virts	T. Virts	J.Williams

Nodes are US modules that connect the elements of the ISS

Node 1, called Unity, was the first US-built element that was launched and connects the US and Russian Segments. Node 1 has 6 ports that provide berthing connections to other modules, ISS infrastructure, and visiting cargo. The module has 4 racks. Some of which are used for stowage to return the cargo back to Earth (ISS_5). Additionally, the dining table (ISS_4) is also located in Node 1

Node 2, called Harmony, connects the US, European, and Japanese laboratories. The module provides docking and berthing ports for Japanese and US vehicles. Node 2 provides crew quarters (ISS_8) for 4 crew members as well as vital functional resources for the operation of the connected elements

Node 3, called Tranquility, is attached to the port side of Node 1 and provides accommodation for life support and exercise equipment. The cupola (ISS_1) is berthed on its nadir (Earth facing) port and provides through multiple windows observation of operations outside the ISS such as robotic activities, the approach of visiting vehicles, and extravehicular activities. Additionally, Node 3 accommodates critical equipment, air revitalization, oxygen, carbon dioxide removal, water recovery system, the waste and hygienic compartment (bathroom; ISS_2), and exercising equipment such as a treadmill (ARED) and a weight-lifting device (ISS_3)

The US laboratory module, called Destiny, is the primary research facility for US payloads. The module hosts 24 equipment racks for accommodation and control of ISS systems and scientific research in physical and biological sciences (ISS_7)

The Permanent Multipurpose Module, called PMM, hosts up to 16 stowage racks (ISS_6) containing equipment, experiments, and supplies, and its additional storage space for bags in the aft endcone

## Cultivable microbial population

**Fig. 2:**
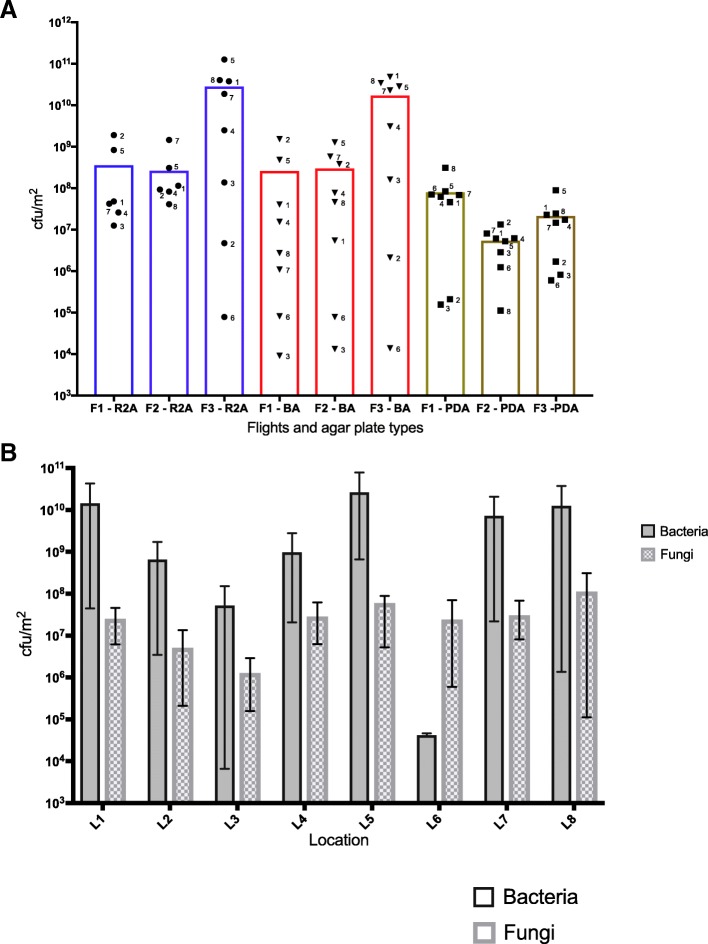
Cultivable bacterial and fungal burden from eight locations on the ISS over a 14-month period. **a** Scatter plot representing the CFU/m^2^ of bacteria and fungi at each location across three flight sampling events. Each column represents a Flight and the type of medium the samples were plated on. Each symbol in that column represents a location sampled during that Flight (*N* = 8). The colored boxes represent the different types of plates the samples were cultured on: Reasoner’s 2A (R2A) or blood agar (BA) plates to isolate bacteria and potato dextrose agar (PDA) plates to isolate fungi. The height of the colored box indicates the average CFU/m^2^ for samples in that group. F1 = flight 1 sampling session, F2 = flight 2 sampling session, and F3 = flight 3 sampling session. NB: There was no growth on R2A plates from location 6 sampled during F1 and F2 and from location 3 sampled during F2. **b** Bar graph representing the CFU/m^2^ based on location. The number of bacteria isolated on R2A and BA plates were averaged to obtain a number for “Bacteria.” The bars represent the average CFU/m^2^ at each location with the capped lines showing the lowest and highest value in that group (*N* = 3). The differences in averages observed in (**a**, **b**) were not statistically significantly different (Kruskal-Wallis test followed by Dunn’s post-hoc test *P* > 0.05). The average number of bacteria and fungi found at each location were similar

The cultivable microbial load from all flight samples and their distribution patterns at various locations are depicted. The average number of bacteria cultured on blood agar (BA) and R2A plates was similar between F1 and F2 but higher at F3 (though this trend was not statistically significant) (Fig. 2a). There were no statistically significant differences in the average bacterial load across the eight locations (Fig. 2b); however, the locations that exceeded 10^10^ CFU/m^2^ during at least one flight sampling event were L1 (port panel next to cupola), L5 (overhead 4), L7 (lab 3 overhead), and L8 (crew quarters), with the lowest counts (less than 10^4^ CFU/m^2^ in at least one sampling event) found at L3 (AREM) and L6 (PMM). Overall, the number of bacteria (combination of R2A and BA growth) isolated from the ISS from all 24 samples ranged from 6.7 × 10^3^ to 7.8 × 10^10^ CFU/m^2^.

Fungi were also cultured from the ISS, ranging from 1.1 × 10^5^ to 3.1 × 10^8^ CFU/m^2^. While, there were no statistically significant differences in fungal load over time, the highest average was found during F1 and the lowest at F2 (Fig. 2a). Similar to what was observed with bacterial counts, no differences in fungal counts were evident across the eight locations (Fig. 2b). When compared to bacteria, the fungal population was lower by 2 to 3 logs at all locations except at L6 where fungal load was 100-fold more than bacterial load (Fig. 2b). Due to the high variability between samples, there were no statistically significant differences overall in the average cultivable counts of bacteria (BA and R2A plates) from all 24 samples compared to the average fungal counts measured from the same 24 samples (*P* > 0.05).

Of the total bacterial and fungal isolates that grew, 133 bacterial isolates and 81 fungal isolates were identified by Sanger sequencing (16S rRNA gene for bacteria; and ITS region for fungi). The bacterial isolates belonged to three phyla: *Actinobacteria*, *Firmicutes*, and *Proteobacteria*. At the genus level, the most predominant genera were *Staphylococcus* (26% of total isolates identified), *Pantoea* (23%), and *Bacillus* (11%) and at the species level, *Staphylococcus aureus* (10%) and both *Pantoea conspicua* (9%) and *Pantoea gaviniae* (9%) (Additional file 1: Figure S1A). Although bacterial counts were similar across all flights (Fig. 2), only members of the family *Enterobacteriaceae* were cultured from F3 samples (Additional file 1: Figure S1A). *S*. *aureus* isolates were tested with the Vitek 2 system (BioMerieux, France) and found to be methicillin-sensitive; however, these isolates were resistant to penicillin, erythromycin, gentamycin, and tobramycin [28]. The whole genomes of 20 biosafety level 2 strains, isolated from these samples, have been sequenced and are publicly available [29].

The fungal population was dominated by *Rhodotorula mucilaginosa* belonging to the family *Sporidiobolaceae* (41% of the 81 examined fungal isolates) and *Penicillium chrysogenum* (15% of 81 fungal isolates) (Additional file 1: Figure S1B). The whole genome of one *Aspergillus fumigatus* strain (isolated from F1, L1 [cupola] sample) was sequenced, its virulence characterized, and this information reported elsewhere [30, 31]**.**

## qPCR-based microbial population

**Fig. 3:**
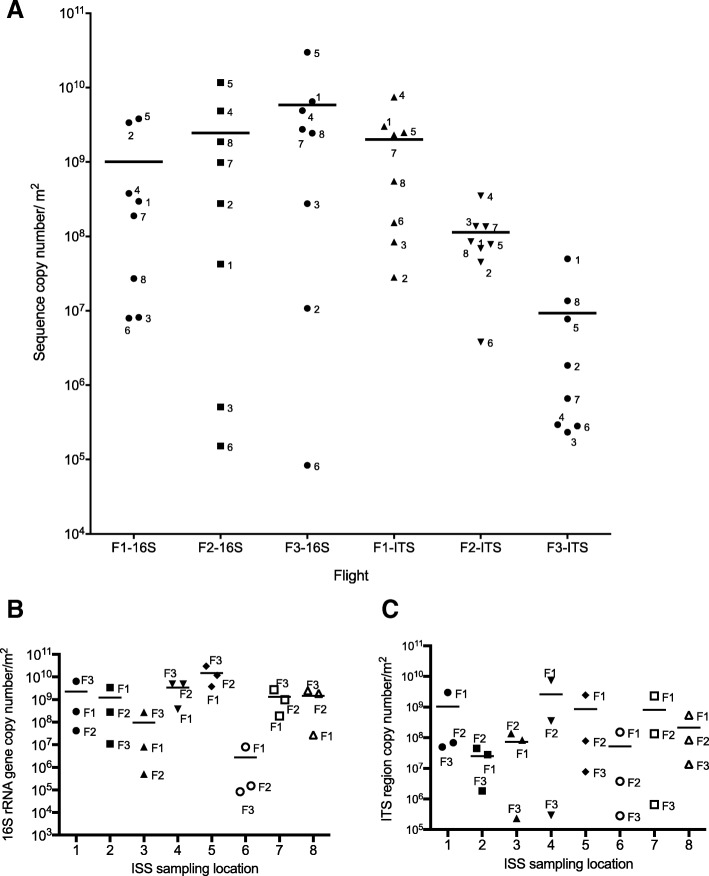
Intact cell membrane/viable bacterial and fungal population aboard the ISS as estimated by PMA-qPCR. **a** Scatter plot comparing the 16S rRNA gene (bacteria) and ITS region (fungi) copy numbers of PMA treated samples collected during flights 1, 2, and 3. Each column represents a single flight and each symbol in a column (labeled with a number) represents one of the eight locations sampled during that flight. The horizontal line in each column represents the average gene copy number/m^2^ for each Flight. **b** Scatter plot comparing 16S rRNA gene and **c** ITS region (fungi) copy numbers across locations. Each column “L” followed by a number represents a location and each dot in a column represents the flight it was sampled from. The horizontal line in each column represents the average copy number/m^2^ at that location. NB: The 16S rRNA gene copy number was not adjusted to the average number per bacterial genome. Control samples were measured and found to be at the level of 10^2^ 16S rRNA gene copies per μL. Even when the initial template volume was increased to 10 μL, the expected 20-fold increase in the gene copy numbers was not observed. In panel **a**, F1-ITS was statistically significantly higher than F3-ITS (*P* < 0.05). No statistically significant differences were observed in panel **b** (*P* > 0.05). The statistical test was performed with the Kruskal-Wallis test followed by Dunn’s post-hoc test

The 16S rRNA gene and the ITS region were targeted in PMA-qPCR to measure intact/viable bacterial and fungal burden, respectively. The changes were not significantly different (*P* > 0.05), although the average number of bacterial 16S rRNA gene copies trended toward increase from F1 to F3. On the other hand, the ITS region amplicons decreased over time with F3 being statistically significantly lower than F1 (Fig. 3a). While the average bacterial (Fig. 3b) and fungal (Fig. 3c) load fluctuated across locations, there were no statistically significant differences in microbial load among different locations. Overall, bacterial loads appeared to be highest at L4 and L5 and lowest at L6, with fungal loads appearing to be highest at L1, L4, L5, and L7 and lowest at L2. The average number of bacteria present on the ISS during this study was 3.1 × 10^9^ 16S rRNA gene copy number/m^2^ and 7.1 × 10^8^ ITS copy number/m^2^ for fungi. A comparison between CFU and gene copy number revealed that on average, 46% of total intact/viable bacteria and 40% of intact/viable fungi could be cultured, while the remainder were viable but yet to be cultured (Additional file 1: Figure S1C).

qPCR was also performed on samples that were not treated with PMA to assess the overall microbial load, which includes intact/viable cells and compromised/dead cells. The average 16S rRNA copy number was 7.1 × 10^9^/m^2^ and the average ITS copy number was 5.1 × 10^8^ m^2^ in these non-PMA-treated samples (Additional file 1: Figure S1D). When the 16S rRNA gene copies were summed up for all locations and all flights, no significant difference was observed with and without PMA (Additional file 1: Figure S1D). This calculation may be affected by the artificial inflation of high copy numbers in samples treated with and without PMA. For example, the highest copy numbers of a sample with 10^10^ copies per m^2^ (for example, Flight 1, Location #2) might mask the samples with 10^7^ copies per m^2^ (for example Flight 1, Location #6). The difference in microbial populations between PMA and non-PMA-treated samples were substantial when the data from individual locations were considered. In general, ~ 0.68% (example: Flight 2; Location #3) to ~ 92.8% (example: Flight 3; Location #5) of the microbial load was present in the PMA-treated sample and therefore considered “viable” (Additional file 1: Figure S1E). Similar reduction in microbial abundance in PMA-treated samples when compared to untreated samples was reported in NASA spacecraft assembly facility (SAF) clean room floors (4 to 21%; [24]), Lunar Mars Analog Habitat floors (10 to 40%; [32]), and HEPA filter particulates of ISS and SAF (1.7 to 66.8%; [2]).

## Bacteriome analysis

**Fig. 4:**
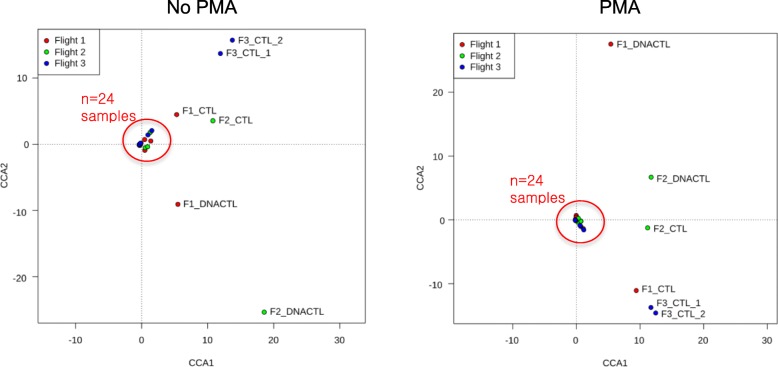
Assessment of bacterial contamination in the ISS environmental samples. Canonical correspondence analysis (CCA) highlighting the differences among species constituents found in samples, treated or untreated with PMA, that were collected from the International Space Station (Flights 1–3) and controls. “DNACTL” represents the DNA extraction control (molecular grade water extracted instead of a sample) and “CTL” represents cloth wipes that were exposed to the environment but not used to sample a surface. F1, F2, F3 denotes the flight

After processing the raw data from 48 samples (24 PMA samples and 24 non-PMA-treated samples) and various wipe and reagent controls, amplicon sequence variants (“ASVs”) (a higher resolution analogue of the ubiquitous “OTU”) [33] that had a cumulative sum of more reads in the controls than the cumulative sum of all the samples, were removed from the dataset. Next, ASVs that were found to be statistically significantly higher (ALDEx2 test, *P* < 0.05) in the control group than in the sample group were further removed from the dataset. Additional file 2: Dataset 1A summarizes the ASV read count in each sample and in each control after the above quality control measures were implemented. Next, the program “SourceTracker” was used to predict what percent of reads in the samples were unique to the samples and what percent were from “contaminating” ASVs (i.e., those were represented in a Dirichlet model trained from the control samples). Additional file 1: Figure S2B summarizes the results from SourceTracker and shows that contamination was negligible in 32 out of 48 samples and for the remaining 16 samples; the contamination was less than 6% of the total sequences. A canonical correspondence analysis (CCA) plot verifies that the bacterial communities of the controls were indeed different than those of the samples (Fig. 4). The ASV table used for downstream analysis, after the above quality control measures were implemented and after verification that the samples represented a unique microbiome, different than that of the controls, is presented in Additional file 2: Dataset S1A.

**Fig. 5:**
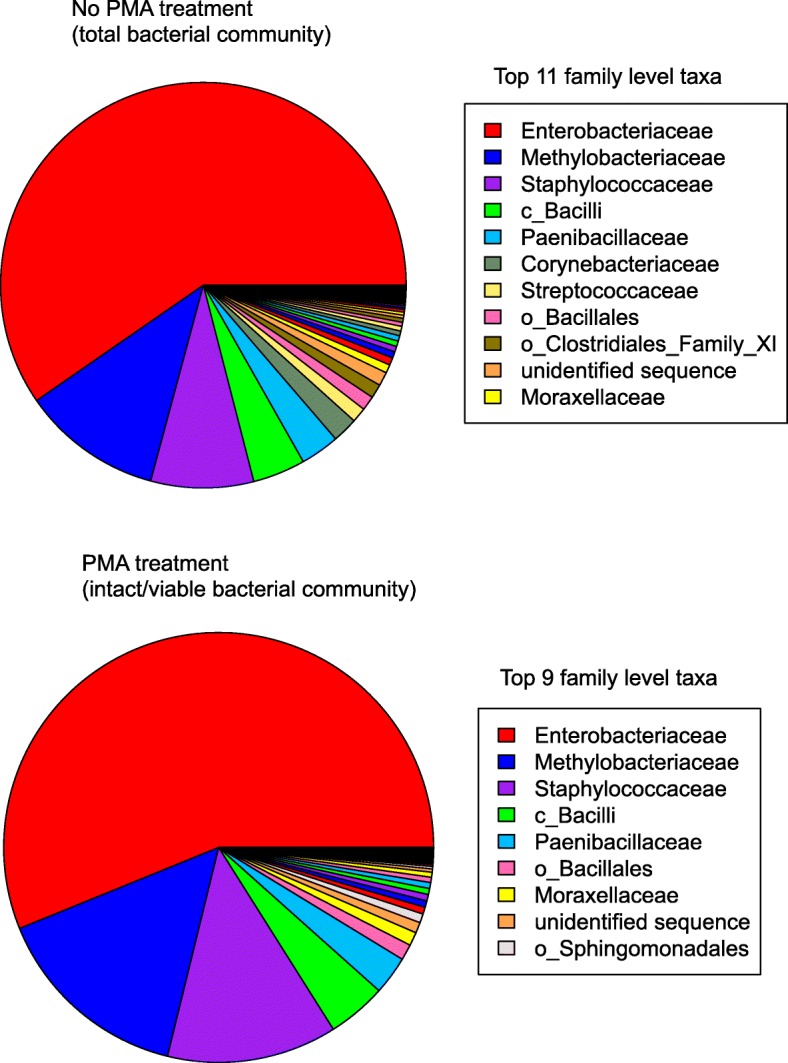
Pie chart showing the relative abundances of taxa identified on the ISS. 16S rRNA gene sequencing was performed on 24 wipes, taken from 8 locations throughout the ISS (see Fig. 1) during 3 flight sampling sessions, spanning 14 months. For each sample, half was treated with PMA (*N* = 24) to detect intact/viable bacteria, while the remaining half was left untreated (*N* = 24) to determine the total bacterial community (both dead cells/cells with a compromised cell membrane and intact/viable). The sequences obtained from both the untreated and PMA-treated samples were summarized to the family level and the relative abundances depicted in this pie chart. In total, 68 different family level taxa were detected but only the most relatively abundant taxa are listed in the legend. A full list of organisms detected can be found in Dataset S1. Those sequences that could not be resolved to the family level are prefixed with either “o” for Order or “c” for Class

A summary of read counts, number of ASVs, and most abundant taxa in the samples are presented in Additional file 3: Table S1. Microbial populations (16S rRNA gene copies) from eight locations over the span of 14 months were calculated from PMA-treated samples (viable/intact bacteria) and non-PMA treated samples (dead/compromised bacteria). Figure 5 shows the proportion of different taxa, summarized to the family level, found in these samples and the variations in their viable populations. In both the PMA- and non-PMA-treated groups, *Enterobacteriaceae* dominated and made up a little over 50% of the sequences detected in all 24 samples combined, followed by *Methylobacteriaceae* (~ 13%) and *Staphylococcaceae* (~ 10%).

**Fig. 6:**
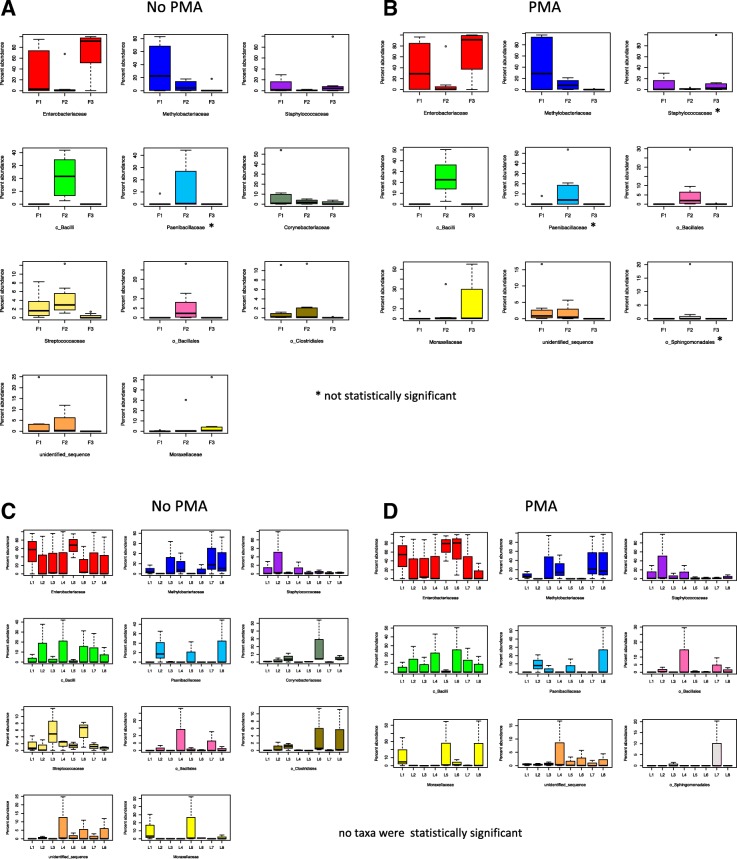
Temporal and spatial distribution of the ISS microbiome over 14 months and across eight locations. Boxplots show the temporal (**a**, **b**) and spatial (**c**, **d**) distribution of the most relatively abundant family level taxa (as presented in Fig. 4). The box in each graph signifies the 75% (upper) and 25% (lower) quartiles and thus shows the percent abundances for 50% of the samples (*N* = 8). The black line inside the box represents the median. The bottom whisker represents the lowest datum still within the 1.5 interquartile range (IQR) of the lower quartile, with the top whisker representing the highest datum still within the 1.5 IQR of the upper quartile. Open circles are outliers. “o” and “c” represent sequences that could not be taxonomically assigned past the order or class level respectively. “F” indicates Flight and “L” indicates Location. **a** Temporal distribution over time in untreated samples. All taxa showed statistically significant changes over time except *Paenibacillaceae* (denoted by *). **b** Temporal distribution in PMA-treated samples. Taxa showed statistically significant changes over time except *Paenibacillacae*, *Staphylococcaceae*, and o_*Sphingomondales* (denoted by *). Spatial distribution in untreated samples (**c**) and in PMA treated samples (**d**). There were no statistically significant differences in these taxa across the eight locations. Significance was measured using ALDEx2 and based on the Benjamini-Hochberg corrected *P* value of the Kruskal-Wallis test (significance threshold, *P* < 0.05). Those sequences that could not be resolved to the family level are prefixed with either “o” for Order or “c” for Class

Of interest was whether the ISS environmental microbiome, and especially the most abundant taxa, changed over time and across locations. The taxa present in the non-PMA-treated (Fig. 6a) and PMA-treated (Fig. 6b) groups showed the same temporal progression: The relative abundances of *Enterobacteriaceae* was highest during F3 and lowest during F2, whereas *Methylobacteriaceae* was the lowest during F3 and highest during F1. *Paenibacillaceae* and members of the class *Bacilli* and order *Bacillales* had high relative abundances during F2, and almost negligible amounts during F1 and F3. Interestingly, F1 and F2 had higher relative abundances of sequences that could not be identified, compared to F3. Statistical analysis using ALDEx2 confirmed that the relative abundances over the three flight sessions were different for all taxa shown, expect for *Paenibacillaceae* in the non-PMA group (Fig. 6a) and *Paenibacillacae*, *Staphylococcaceae*, and *Sphingomondales* in the PMA-treated group (Fig. 6b).

Unlike the differences observed over time, no statistically significant differences in relative abundances were observed between the eight locations (Fig. 6c, d); however, some interesting trends are worth noting: *Enterobacteriaceae* was well represented at each location, with the highest relative abundance observed at L1, L5, and L6. *Methylobacteriaceae* was also highly represented across locations except for L2, L5, and L6.

The barplot in (Additional file 1: Figure S3) provides a more detailed representation of the relative abundances of family level taxa in each sample. Upon visual inspection, it appears that bacterial diversity within a sample was the lowest during Flight 3, which consisted predominately of *Enterobacteriaceae*, and was the highest during F2. This observation was statistically confirmed (*P* < 0.05) by calculating (i) Shannon’s diversity index, which measures both taxa presence and relative abundance and (ii) taxon richness, which reports the number of unique taxa in a sample (Additional file 1: Figure S4A and B). Noteworthy, both alpha diversity (measured with Shannon’s diversity index and taxa richness) and beta diversity (measured with ALDEx2) showed no differences between PMA and non-PMA treated samples across all three flights, suggesting that the DNA recovered from the ISS were from intact/viable organisms.

When the ASVs were summarized to the genus level, 121 taxa were detected, 77 of which could be assigned to known genera (Additional file 1: Figure S5). Of those 77 genera, 68% of them are known constituents of the human microbiome and the remaining 32% are found in environments such as soil and water.

## Mycobiome analysis

Amplicon sequencing of the fungal ITS region was performed on samples collected during F1 and F2. Since F3 exhibited low abundance of cultivable fungal counts, it was not possible to generate amplicons for further sequencing. Similar to what was done with bacterial sequences, OTU counts that were higher across controls compared to samples were removed from the dataset. For one OTU, even though the cumulative read count of the controls was 33 times lower than the samples, since the count was 300,000, it was removed from the dataset. Additional file 2: Dataset S1B shows the fungal OTU table that was used for analyses after OTUs associated with controls were removed. The SourceTracker results for the mycobiome are shown in Additional file 1: Figure S6A and the total OTU read count for the sample and control wipes is presented in Additional file 1: Figure S6B.

The fungal population consisted of four genera plus members belonging to one phylum, two classes, and four families, in addition to sequences that could not be classified (Additional file 1: Figure S7). The temporal and spatial distribution of the five most relatively abundant taxa are shown in Additional file 1: Figure S8. With the exception of *Sporidiobolaceae* which was higher in F2 compared to F1, there were no other statistically significant differences in fungal taxa between flights.

Unlike bacteria, fungal diversity within samples (i.e., alpha diversity) did not change between Flight 1 and 2 (Additional file 1: Figure S4C and D). Similar to what was observed with bacteria, alpha and beta diversity were similar between the PMA- and non-PMA treated samples across these two flights.

## Comparison of ISS environmental microbiome with Earth microbiome

**Fig. 7:**
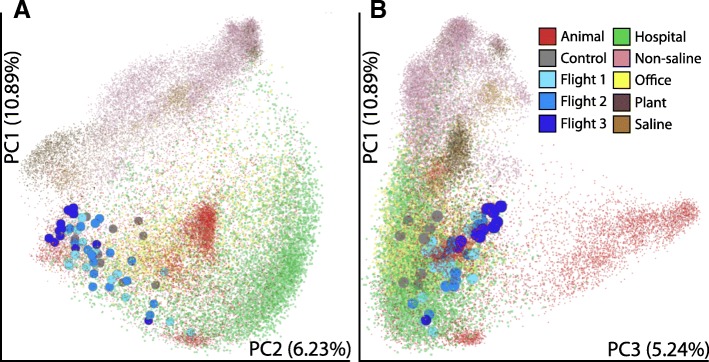
Comparison of ISS environmental microbiome with microbiomes of Earth. Principal coordinates analysis of unweighted UniFrac distances from the Earth Microbiome Project [96], the Hospital Microbiome Project ([5], Qiita study 10,172), and the Office Succession Study [105] depicting **a** PC1 vs. PC2 and **b** PC1 vs. PC3. The Hospital Microbiome Project and Office Succession Study are composed predominantly built environment samples (e.g., walls, floors, etc.). All three ISS flight sample sets group with the built environment samples. The primary separation along PC1 is environmental or plant associated samples vs. animal surface, secretion, or built environment. The primary separation along PC3 is whether a sample is associated with the animal gut

Publicly available sequences of PMA-treated samples collected from two JPL clean rooms, ISS dust, ISS HEPA filters, and surface samples from an inflated Lunar Mars analogue habitat (ILMAH) were compared. As is clear from the PCoA plot shown in Additional file 1: Figure S9, the ISS surface microbiome is a unique microbiome, differing from the ISS-dust, ISS-HEPA, JPL clean rooms, and Lunar/Mars-like human-occupied habitats. In addition, PMA-untreated microbial diversity associated with ISS environments was compared to results obtained from the Earth Microbiome Project, hospital environment, and office spaces. The ISS samples grouped with the built environment data, as shown in Fig. 7. This relationship also suggests that, as expected, the environmental locations sampled on the ISS harbored microbes more similar to those on animal surfaces (e.g., skin) than to environmental soil samples. Similar pattern was seen with built environmental samples from Earth. Thus, the ISS samples resembled other Earth built environment samples, and the differences among flights (including differences in DNA extraction and PCR amplification) were relatively small compared to the differences among different sample types.

Using the unrarefied data, the unique sub-operational taxonomic units (sOTUs) present in the ISS data were also compared with other built environment datasets collected on various locations of Earth to determine whether any sOTUs appear to be unique to the ISS. For this analysis, only Flight 3 data were included since all the other built environment datasets used the same primer set. The sOTUs observed in the ISS controls were removed. There were four sOTUs that appear to be unique relative to all the built environment datasets analyzed (see Additional file 4: Table S4), although they accounted for a very small total amount of the sequence mass (~ 0.0005% of the reads). These unique sOTUs exhibited high identity to *Bacteroides* sp., *Gottschalkia acidurici*, *Paenibacillus thailandensis*, and *Thermus thermophilus* based on BLAST to nr/nt [34]. One single sOTU belonging to *T*. *thermophilus* was unique in Flight 3 samples and was not observed in the Earth Microbiome Project, nor in other built environment datasets on Earth.

## Discussion

The ISS environmental microbiome was characterized from eight locations throughout the ISS during three flight sampling events over a period of 14 months. This allowed the examination of temporal and spatial distribution of microbial populations on the ISS. This is the first study to utilize culture, qPCR, and amplicon sequencing to study the surfaces of the US segment and revealed a diverse intact/viable population of bacteria and fungi that changed over time but were similar across locations.

Several studies have been carried out to measure microbiological cleanliness of the ISS environment using cultivation-based approaches since the inception of this closed system [35]. Recently, several ISS surfaces in the US nodes [36] and Japanese Kibo module [37] were swabbed and targeted amplicon sequencing carried out. However, these studies did not measure the intact/viable microbiome which therefore could not be compared to culture counts nor provide an assessment for crew risk. Previous reports on the ISS intact/viable microbiomes on the ISS were only examined from air filters and debris collected via vacuum cleaner bag [2]. The ISS is a unique environment and one question that is of interest to many is how this intact/viable microbiome compares to other closed, regulated environments (Additional file 1: Figure S9 and Fig. 7). The ISS environmental microbiome resembles that of animal skin surfaces rather more than the soil microbiome. This might be due to the fact that cargo sent to the space station was cleaned thoroughly, and hence soil-associated microorganisms were not present.

The predominant organisms on ISS surfaces consisted of those that are associated with humans, with some considered opportunistic pathogens on Earth. As to whether they could cause disease in astronauts on the ISS is unknown, as it would depend on the health status of each individual and how these organisms function while in the space environment. Regardless, the detection of possible disease-causing organisms highlights the importance of further genomic and transcriptomic studies to examine how these ISS microbes function in space and how they may impact astronauts’ health. Correlating viable but opportunistic pathogens with crew member health is likely to raise too many questions about access to the crew microbiome data which is not yet publicly available, and about statistical power: because the ISS has few occupants and high turnover, identifying statistically relevant trends in crew member health that correlate with environmental microbiomes is not possible. From the time the ISS was built in 1998, as of Aug 3, 2017, 222 astronauts visited the ISS, and microbial signatures left behind by previous astronauts might interfere with the predictions. Consequently, the present ISS environmental microbial metrics could not be linked to any particular crew member. Since there were no differences in community composition and richness between PMA- and non-PMA treated samples, it suggests that the DNA analyzed from these possible opportunistic pathogens residing on the ISS environmental surfaces are indeed intact/viable and not dead organisms.

Noteworthy, approximately 46% of intact/viable bacteria and 40% of intact/viable fungi could be cultured with the culture media used during this study. This percentage is high when compared to spacecraft assembly cleanrooms on Earth where only 1 to 10% of intact/viable microorganisms can be cultured [38]. The possible explanation is that the ISS is not deprived of nutrients like spacecraft assembly cleanrooms, and while this is a hermetically sealed environment, it is exposed to microbes from astronauts (maximum six astronauts at a given time) and cargo (delivered ~ 4–6 times per year). Furthermore, no relationship was found between microbial load and sample processing time (F1: 7 days, F2: 9 days, and F3: 6 days).

Many of the organisms detected on the ISS are known to form biofilms that belong to both bacterial (*Acinetobacter*, *Sphingomonas*, *Bacillus*, *Burkholderia*, *Corynebacterium*, and *Klebsiella*) [39] and fungal (*Penicillium*, *Aspergillus*, *Cryptococcus*, and *Rhodotorula*) [40] genera. This could pose problems for astronauts if they do become infected as biofilms are known to promote resistance to antibiotics [41]. Also, biofilm formation on the ISS could decrease infrastructure stability by causing mechanical blockages, reducing heat transfer efficiency, and inducing microbial influenced corrosion [42]. Some of the microorganisms that were identified on the ISS that have been implicated in microbial-induced corrosion on Earth are *Methylobacterium*, *Sphingomonas*, *Bacillus*, *Penicillium*, and *Aspergillus* [43–46]; however, the role they play in corrosion aboard the ISS remains to be determined. Elucidating the potential ability to form biofilms and the magnitude of actual biofilm formation on ISS surfaces is important during long-term space missions to maintain structural stability of the crew vehicle when routine indoor maintenance cannot be as easily performed.

As expected, culture-based analysis did not detect as many genera as that with amplicon sequencing; however, its importance should not be overlooked as species level identity of ISS microbial constituents could be obtained when isolates were available. Furthermore, isolating organisms allowed us to conduct a separate study to examine the influence of microgravity and radiation on antibiotic resistance and virulence [47] and to obtain whole genome sequences of organisms that grow in space, for future comparative genomics [29]. Similar to a previous study on ISS HEPA filters, where the novel organism, *Solibacllus kalamii* [48]was able to be identified only through culture analysis, this study has also allowed us to detect a recently identified novel species *Enterobacter bugandensis* that was associated with human disease on Earth [49, 50]*.* A high percentage of the cultivable population represented opportunistic pathogens such as *S*. *aureus*, *Staphylococcus hominis*, *Staphylococcus haemolyticus*, *P*. *conspicua*, *Acinetobacter pittii*, *Klebsiella quasipneumoniae*, and *A*. *fumigatus*. This could have potential health impacts on astronauts, as bacteria and fungi have been shown to be transferred between surfaces and humans upon contact [51]. The scope of the present study was not to determine whether these microorganisms were more virulent or resistant to antibiotics than on Earth but the whole genome sequences have been published for the isolated biosafety level 2 microorganisms [29] and comparative genomics of these ISS isolates with Earth strains is now possible for further investigation.

Members of the family *Staphylococcaceae* and *Enterobacteriaceae* were the most predominant organisms on ISS surfaces of the US module, similar to what has been published for the Japanese module of the ISS [52], and were detected in almost every sample by both culture and amplicon sequencing (Additional file 1: Figure S10). Both are human-associated organisms, with *Staphylococcaceae* commonly found on the skin and in the nasal passage, and *Enterobacteriaceae* commonly associated with the gastrointestinal tract. These two taxa are also abundant in fitness centers [53], office buildings [54], and hospitals [5], suggesting that the ISS is similar to other built environments on Earth, in that its microbiome is shaped by human occupation [32]. On Earth, it has been observed that incoming intensive care unit (ICU) patients have a significantly higher risk of acquiring infections if the previous occupant was a carrier, despite terminal cleaning of the bed and the room [55–57]. Thus, habitation of the same area, regardless of whether individuals interact or not, may contribute to disease spread. Further studies assessing how long organisms survive on ISS surfaces and how readily they can be passed on from one individual to another in space can lead to the development of countermeasures to minimize the spread of infections from one astronaut to another during simultaneous or even separate flight missions.

*Methylobacteriaceae*/*Methylobacterium* was also dominant across the ISS and could be cultured from several samples. This is a hardy organism that can withstand harsh conditions, such as ionizing radiation and strong cleaning detergents and has previously been found in NASA spacecraft assembly clean rooms [58], hospital ICUs [59], and the MARS500 habitat [60]. *Moraxallaceae*, another abundant organism on the ISS, also thrives in harsh conditions, being present in higher relative abundances in spacecraft assembly cleanrooms [61], areas of the home that utilize a lot of chemicals (i.e., washing machine) [62], and deep sea sediment of inactive hydrothermal vents [63].

*R*. *mucilaginosa* was the predominant fungal isolate from the culture analysis, and belongs to the *Sporidiobolaceae* family which was found in high relative abundances across the ISS with amplicon sequencing. This organism can survive inside dishwashers despite high temperatures and chemical exposure [64].

Numerous studies conducted on Earth have shown that the type and amount of human activity in a particular location impacts that indoor microbiome [65–67] and while there were no differences in the average microbial load (by culture and qPCR) nor community structure (amplicon sequencing), it was clear that there were variations between sampling points across different locations. Among the eight locations sampled (Table 1), location #6 (permanent multipurpose module [PMM] port 1) exhibited low concentrations of cultivable (Fig. 2), viable (Fig. 3a), and total (data not shown) microbial burden. The PMM is a specific location within the Node 1 Nadir module of ISS (Fig. 1) to store bags intact as opposed to open and place them into racks. Minimum crew activities are expected in PMM location #6 and hence microbial abundance might be minimal compared to other locations that are heavily occupied by astronauts in a day to day activities such as observing window cupola (location #1), toilet (location #2), ARED exercise platform (location #3), dining table (location #4) performing several experiments, or sleeping quarters (location #8). In a study performed by Mayer et al. [32] in an inflated lunar/Mars analog habitat, the cultivable bacterial load was in the range of 10^3^–10^5^ per m^2^ after the student crew inhabited the analog station. There were no significant changes in microbial load between the more active areas like the laboratory and other locations, while bedroom cultivable bacterial load increased toward the end of 30-day occupation. The fungal cultivable population was lower than bacterial, but it was in the range from 10^2^ to 10^4^ per m^2^ [68]. In contrast, the ISS results showed that cultivable microbial load were not uniform between locations and warrant more study.

In general, temporal differences were observed within the bacterial population: F2 samples had higher microbial diversity (alpha diversity) than F1 and F3 samples; only *Enterobacteriaceae* were cultured from F3 samples and nine out of the ten most relatively abundant family level taxa differed over the three flights. These temporal differences may be due to the different occupants onboard the ISS during each of the flight sampling session. Earth indoor microbiome studies have shown that humans shed microbes to indoor surfaces upon contact, playing a pivotal role in shaping the indoor microbiome [17]. Similarly, a study of the inflatable Lunar/Mars analog conducted here on Earth showed differences in bacterial communities between day 0 (before human occupation) and after 30 days of habitation [32] showing the effects of human occupation on indoor microbial communities in a space-like environment. Of the nine astronauts that were present aboard the ISS from F1 to F2 (2 months apart), only three were present during both flights and none of the astronauts present during F1 or F2 were on the ISS during F3 sampling. Further studies that collect microbial information from astronauts in parallel with air and surface samples would help elucidate how much of an impact astronaut have toward the ISS microbiome. Unlike bacteria, fungal communities were stable over time with no temporal differences, and this could be due to the fact that fungal and bacterial communities follow different environmental determinants [69].

It should be noted that F3 samples were sequenced separately from the F1/F2 samples and used different but similar V4 primers (see “Materials and methods” section for more details). Due to the SpaceX-7 launch failure and the uncertainty of when F3 sampling kits would be flown to the ISS for sampling, it was not possible to sequence F3 with F1/F2. However, the samples were collected in the same manner, processed identically, and the same protocol used for DNA extraction. We do not believe that the choice of primers, nor the separate sequencing runs, have influenced the differences in temporal distribution presented in this manuscript for the following reasons: (i) Additional file 5: Table S2 shows the organisms that were statistically significantly different over time and shows the efficiency of each primer pair in detecting these organisms, which are almost identical. (ii) A metagenomics analysis was performed using the same DNA samples as for amplicon sequencing and all three flights were sequenced simultaneously and without multiple displacement amplification prior to sequencing. The family level barplot for this metagenomics data in Additional file 1: Figure S11 shows the same pattern distribution of taxa, as presented in Additional file 1: Figure S3 for the amplicon sequencing. (iii) Lastly, all statistical analyses were performed with ALDEx2 which estimates per-feature technical variation within each sample using Monte-Carlo instances drawn from Dirichlet distributions. ALDEx2 uses the centered log-ratio transformation that ensures that data are scale invariant and sub-compositionally coherent meaning that all samples are numerical consistent with each other, regardless of the total sequencing read capacity at the time of sequencing [70, 71]. This ensures that the statistical results are robust and are not influenced solely by the differential detection of ASVs that can occur during different sequencing runs.

Many 16S rRNA and ITS sequences could not be identified via amplicon-targeted analyses, but a metagenomics approach recently conducted identified 318 bacterial and fungal species in these samples [72]. In addition, shotgun metagenome analysis carried out by Singh et al. [72] from the same samples revealed that reads associated with carbohydrate metabolism, amino acid derivatives and cofactors, vitamins, etc. were the highest among all three flights. Similarly, computational analyses showed that the *Legionella* resistome, cobalt-zinc-cadmium resistance, and multi-drug resistant resistance efflux pump were high on all flights and all locations. The shot-gun reads associated with antimicrobial resistant genes in Flight 3 increased by twofold when compared with Flights 1 and 2 which also predicted the persistence of opportunistic pathogens in Flight 3 samples [72]. Collective beta-Lactam resistance derived from the metagenome sequence analysis shows that physical (*OmpF*, *OmpC*), transformational (penicillin-binding protein), and degradational (*AmpC*), and MDR efflux pump (OMP, RND, MPF) mechanisms were allocated by the microorganisms on the ISS [72].

Exploring the spatial and temporal distribution of intact/viable microbial populations of closed systems such as the ISS will facilitate planning of future human habitation of Moon, Mars, and beyond. Accumulation of intact/viable microbial cells in a confined environment poses a health risk to all inhabitants. This study on bacterial and fungal load and diversity across the ISS provides a comprehensive catalog of what can be found in closed space systems and can be used to develop safety measures for NASA to meet the requirements for long-term space travel or living in space. The implications of this study are not only limited to space biology but can have significant impact on cleanrooms here on Earth such as those in the pharmaceutical and medical industries.

## Materials and methods

### Sample kit preparation and sample collection

Sampling wipes were prepared at the Jet Propulsion Laboratory (JPL; Pasadena, CA). Briefly, each polyester wipe (9″ × 9″; ITW Texwipe, Mahwah, NJ) was folded two times and soaked in 15 mL of sterile molecular grade water (Sigma-Aldrich, St. Louis, MO) for 30 min followed by the transfer to a sterile zip lock bag [73]. The sampling kit was assembled at NASA Ames Research Center (ARC, Moffett Field, CA). The implementation team at NASA ARC delivered the kit to the Cargo Mission Contract at Johnson Space Center (Texas) which was then transferred to Kennedy Space Center (Florida) in order to be loaded into the Space Exploration Technologies (SpaceX) Dragon spacecraft prior to launch. Each sampling kit was sent to the ISS onboard the SpaceX-5, -6, -8, rockets and returned to the Earth onboard the Russian vehicle (Soyuz TM-14) and Dragon capsule (SpX-6 or -8). Eight different locations were sampled on the ISS using the polyester wipes described above (see Fig. 1 for a summary of the sampling locations). The metadata associated with the samples and collections is summarized in Additional file 6: Table S3.

The study requirements stated that there should be no cleaning at least 4 days prior to sampling. When the cleaning occurred during the weekends, it was done at the crew’s discretion without suggestions about the specific locations, therefore following the typical routine of activities on the ISS. The disinfectant wipes that are used in the ISS contain octyl decyl dimethyl ammonium chloride (0.0399%), dioctyl dimethyl ammonium chloride (0.01995%), didecyl dimethyl ammonium chloride (0.01995%), alkyl (50% C14, 40% C12, 10% C16) dimethylbenzylammonium chloride, and dimethylbenzylammonium chloride (0.0532%). During each flight, one astronaut performed all the sampling and used the wipes to sample one square meter. A new pair of individually packed sterile gloves (KIMTEC Pure G3 White; Nitrile Clean-room Certified; Cat. HC61190) were used before sampling the next location. The crew was instructed to collect samples from the same surfaces during all three sampling sessions. A control wipe (environmental control) was taken out from the Zip lock bag, unfolded, waved for 30 s, and packed back inside a new sterile zip lock. One control wipe was included for each flight session. Similarly, an unused wipe that was flown to the ISS and brought back to Earth along with the samples served as a negative control for sterility testing. If field controls (wipes that were exposed to the ISS environment but not used in active sampling) showed any signs of microbial growth, then negative controls would be assayed for cultivable counts to check sterility of the wipes used for sampling. However, none of the field controls showed any CFUs for all three flights. The samples were stored at room temperature in orbit. After sample collection, samples were returned to Earth after 7 days for Flight 1, 9 days for Flight 2, and 6 days for Flight 3. The kits were delivered to JPL immediately after arrival to Earth at 4 °C with processing at JPL commencing within 2 h of receipt.

### Sample processing

Sample processing took place in a ISO 7 (10K class) cleanroom at JPL. In a certified biosafety cabinet, each wipe was aseptically removed from the zip lock bag and transferred to a 500 mL bottle containing 200 mL of sterile phosphate-buffered saline (PBS; pH 7.4). The bottle with the wipe was shaken for 2 min followed by concentration with a Concentrating Pipette (Innova Prep, Drexel, MO) using a 0.22 μm Hollow Fiber Polysulfone tips (Cat #: CC08022). Each sample was concentrated to 4 mL with PBS elution fluid (Cat #). Then, 3 mL of this concentrated sample was split into two 1.5 mL aliquots. One aliquot was treated with PMA (18.25 μL of 2 mM PMA, resulting in a final concentration of 25 μM) to assess cells that were viable or had an intact cell membrane [24], while the second aliquot was handled in a similar manner but without the addition of PMA. The PMA and non-PMA-treated aliquots were incubated in the dark at RT for 5 min, followed by 15 min of photoactivation using the PMA-Lite™ LED Photolysis Device, specifically designed for photoactivation of PMA (Biotium, Hayward, CA). The PMA- and non-PMA -treated aliquots were then split into two 0.75 mL aliquots. One aliquot was transferred to bead beating tubes containing Lysing Matrix E (MP Biomedicals, Santa Ana, CA), followed by bead beating for 60 s using the vortex sample holder (MO Bio, Carlsbad, CA). The bead-beaten aliquot and the aliquot without bead beating were combined for their corresponding PMA-treated and non-treated samples. DNA extraction was performed with the Maxwell 16 automated system (Promega, Madison, WI), in accordance with manufacture instructions using the Maxwell 16 Tissue LEV Total RNA purification kit. A Maxwell control (MC) without any sample added in its cartridge was run concurrently with each flight sample. The extracted DNA was eluted in 50 μL of water and stored at − 20 °C until further analysis.

### Estimation and identification of cultivable microbial population

The concentrated samples were diluted in PBS (up to 10^−6^ of each original sample) and 100 μL of each dilution was plated (in duplicate) on Reasoner’s 2A agar (R2A for environmental bacteria), Potato Dextrose Agar with chloramphenicol (100 μg/mL; PDA for fungi), and blood agar (BA for human commensals; Hardy Diagnostics, Santa Maria, CA). R2A and PDA plates were incubated at 25 °C for 7 days and BA plates at 35 °C for 2 days at which time colony forming units (CFU) were calculated. Whenever possible, a minimum of five isolates of distinct morphologies were picked from each plate, from each ISS sampling location. The isolates were then archived in semisolid R2A or PDA slants (agar media diluted 1:10) and stored at room temperature. Once a culture was confirmed to be pure, two cryobead stocks (Copan Diagnostics, Murrieta, CA) were prepared for each isolate and stored at − 80 °C. A loopful of purified microbial culture was directly subjected to PCR and the targeted fragment was amplified (colony PCR), or DNA was extracted with the UltraClean DNA kit (MO Bio, Carlsbad, CA) or Maxwell Automated System (Promega, Madison, WI). The extracted DNA was used for PCR to amplify the 1.5 kb 16S rRNA gene in order to identify bacterial strains. The following primers were used for the 16S rRNA gene amplification: the forward primer, 27F (5′-AGA GTT TGA TCC TGG CTC AG-3′) and the reverse primer, 1492R (5′-GGT TAC CTT GTT ACG ACT T-3′) [74, 75]. The PCR conditions were as follows: denaturation at 95 °C for 5 min, followed by 35 cycles consisting of denaturation at 95 °C for 50 s, annealing at 55 °C for 50 s, and extension at 72 °C for 1 min 30 s and finalized by extension at 72 °C for 10 min. The ITS region was amplified using the forward primer ITS1F (5′-TTG GTC ATT TAG AGG AAG TAA-3′) [76] and reverse primer Tw13 (5′-GGT CCG TGT TTC AAG ACG-3′) [77] to obtain a ~ 1.2 kb product. The PCR conditions were as follows: initial denaturation at 95 °C for 3 min followed by 25 cycles of 95 °C for 50 s, annealing at 58 °C for 30 s, and extension at 72 °C for 2 min, followed by a final extension at 72 °C for 10 min. The amplicons were inspected on a 1% agarose gel. When bands for products were visible, amplification products were treated with Antarctic phosphatase and exonuclease (New England Biolabs, Ipswich, MA) to remove 5′- and 3′-phosphates from unused dNTPs before sequencing. The sequencing was performed by Macrogen (Rockville, MD) using 27F and 1492R primers for *Bacteria*, and ITS1F and Tw13 primers for *Fungi*. The sequences were assembled using SeqMan Pro from DNAStar Lasergene Package (DNASTAR Inc., Madison, WI). The bacterial sequences were searched against EzTaxon-e database [78] and the fungal sequences against the UNITE database [79]. The identification was based on the closest percentage similarity (> 97%) to previously identified microbial type strains.

### qPCR assay

Following DNA extraction with the Maxwell Automated system, quantitative polymerase chain reaction (qPCR), targeting the partial 16S rRNA gene (bacteria) or partial ITS region (fungi), was performed with SmartCycler (Cepheid, Sunnyvale, CA) to quantify the microbial abundance. Primers targeting the partial 16S rRNA gene were 1369F (5′-CGG TGA ATA CGT TCY CGG-3′) and modified 1492R (5′-GGW TAC CTT GTT ACG ACT T-3′) [80]. Primers targeting the ITS region were NS91 (5′-GTC CCT GCC CTT TGT ACA CAC-3′) and ITS51 (5′-ACC TTG TTA CGA CTT TTA CTT CCT C-3′) [81]. Each 25-μL reaction consisted of 12.5 μL of 2X iQ SYBR Green Supermix (BioRad, Hercules, CA), 1 μL each of forward and reverse oligonucleotide primers (10 μM each), and 1 μL of template DNA (PMA treated and non-treated samples). Each sample was run in triplicate; the average and standard deviation were calculated based on these results. Purified DNA from a model microbial community [82] served as the positive control and DNase/RNase free molecular-grade distilled water (Promega, Madison, WI) was used as the negative control in each run. The reaction conditions were as follows: a 3-min denaturation at 95 °C, followed by 40 cycles of denaturation at 95 °C for 15 s, and a combined annealing and extension at 55 °C for 35 s. The number of gene copies in the samples were determined by running a standard curve, which was generated using serial dilutions (10^8^–10^2^) of *Bacillus pumilus* SAFR-032 16S rRNA gene as described previously [2]. The qPCR efficiency was ~ 98% for each run. The negative control values were not deducted since the values were at ~ 100 copies per 1 or 10 μL and not scalable (yielded the same results despite using 1 μL and 10 μL of DNA templates was used).

### Illumina sequencing - Bacteria

#### Flight sampling 1 and 2

Bacterial diversity was assessed by analyzing the V4 hypervariable region of the 16S rRNA gene coding sequence. Amplification was performed with the following primer pair: forward primer, A519F (new nomenclature: S-D-Arch-0519-a-S-15), 5′-CAG CMG CCG CGG TAA-3′, and the reverse primer 802R (new nomenclature: S-D-Bact-0785-b-A-18,) 5′-TAC NVG GGT ATC TAA TCC-3′ [83]. Expected amplicon size is 283 for Bacteria as estimated for 16S rRNA gene sequences deposited in the Silva SEED Reference Database [84].

Fungal diversity was assessed by analyzing the ITS1 region between 18S and 5.8S rRNA coding sequences. Amplification primers were ITS1-F_KYO2 (5′-TAG AGG AAG TAA AAG TCG TAA-3′) and ITS2_KYO2 (5′-TTY RCT RCG TTC TTC ATC-3′) [85]. Expected amplicon length distribution is 271 ± 90 bp for *Ascomycota*, 284 ± 42 bp for *Basidiomycota*, and 216 ± 94 bp for non-*Dikarya* species [86].

PCR synthesis of SSU-V4 and ITS1 amplicons was performed using Q5 High-Fidelity PCR Kit (New England Biolabs, Ipswich, MA) according to the manufacturer’s instructions. The 40-μL reaction mixtures were incubated under the following conditions: initial denaturation at 94 °C for 3 min followed by 35 cycles of 94 °C for 30 s, 47 °C for 30 s, and 72 °C for 90 s, with a final extension at 72 °C for 5 min. Afterwards, each reaction mixture was fractionated by electrophoresis on 2% agarose gel, recovering all PCR products in the size range of 200 to 400 bp. The amplicons were isolated from gel slices using silica spin-columns [87], and eluted with nano-pure water. The purified amplicons were tagged with barcoded Illumina adapters using TruSeq DNA PCR-Free Library Prep Kit LT (Illumina, San Diego, CA) according to the manufacturer’s instructions. The libraries were quantified on a TBS-380 Fluorimeter (Turner BioSystems, Sunnyvale, CA) using PicoGreen dye (Invitrogen, Carlsbad, CA) as a dsDNA-binding fluorogenic reagent. The dsDNA length distribution in individual library preps was assessed by analysis on a 2100 Bioanalyzer with High Sensitivity DNA chip (Agilent Technologies, Santa Clara, CA). The libraries were pooled to be present at equimolar concentrations in each mixed sample with total concentration of 10 nM. The first mixed sample contained 20 16S rRNA-V4 libraries and 17 ITS1 libraries representing the first ISS sampling session together with corresponding controls. The second mixed sample contained 21 16S rRNA-V4 libraries and 20 ITS1 libraries representing the second ISS sampling session and corresponding controls. The two sample sets were sequenced on a NextSeq 500 Sequencing System (Illumina, San Diego, CA) with NextSeq 500/550 Mid-Output v2 Kit for 300 main and 6 index cycles.

#### Flight sampling 3

DNA from these samples was amplified using 1 μL of gDNA in triplicate 25 μL reactions using Platinum Hot Start PCR master mix (Thermo Fisher cat# 13000012) and custom golay barcoded primers of the 16S V4 region, 515fB (5′-GTG YCA GCM GCC GCG GTA A-3′) and 806rB (5′-GGA CTA CNV GGG TWT CTA AT-3′), (expected amplicon size ~ 291 bp) as described in the http://www.earthmicrobiome.org for 94 °C 3 min and 35 cycles at 94 °C 45 s, 50 °C 60 s, 72 °C 90 s followed by 72 °C 10 min and held at 4 °C. Triplicate reactions were then pooled into a single tube and quality assessed. The amplicons were run on a 2% agarose gel and quantified using PicoGreen to access quality and relative quantity. All samples were pooled in equal volume into a single tube and then processed through the MoBio PCR cleanup kit to remove adaptors and primers. Final cleaned pools were then sequenced on a HiSeq 2500 2 × 150 bp Rapid Run.

### Illumina sequence processing—Bacteria (flight 1, 2, and 3)

For F1 and F2 samples, the forward reads were de-multiplexed by using fastq-multx v. 1.02.772, a tool from ea-utils software package [88], with the forward amplification primers for prokaryotes as search targets. The reads were further processed to remove all remaining sequences of the amplification primers and the Illumina TruSeq adapters from their 3′-ends using consecutively fastq-mcf v. 1.04.807 program [88] for exact sequence search, and agrep (http://www.tgries.de/agrep/) and treagrep (0.8.0: https://github.com/laurikari/tre/) programs for search allowing up to three mismatches between the primers/adapters and the reads to accommodate for sequencing errors. The F3 reads were demultiplexed and adaptors removed using Qiita (http://qiita.ucsd.edu) using the parameters max_barcode_errors: 1.5; barcode_type: golay_12; and phred_quality_threshold: 3.

The demultiplexed reads for F1, F2, and F3 were then processed using the DADA2 pipeline, trimming the 3′ end of the forward reads to a length of 130  bp, and setting the filter parameters to maxN = 0, maxEE = 2, trunQ = 2, and rm.phix = True. The DADA2 pipeline (https://benjjneb.github.io/dada2/index.html) was followed to obtain an amplicon sequence variant table (“ASV” table), a “higher resolution analogue of the ubiquitous OTU table”. Taxonomy was assigned used the SILVA reference database.

### Illumina sequence processing—Fungi (Flight sampling 1 and 2)

The forward reads were de-multiplexed by using fastq-multx v. 1.02.772, a tool from ea-utils software package [88], with the forward amplification primers fungi as search targets.

The 5′-ends of the sorted reads were trimmed for a predetermined length based on the length of the corresponding amplification primer for each dataset. The reads were further processed to remove all remaining sequences of the amplification primers and the Illumina TruSeq adapters from their 3′-ends using consecutively fastq-mcf v. 1.04.807 program [88] for exact sequence search, and agrep (http://www.tgries.de/agrep/) and treagrep (0.8.0: https://github.com/laurikari/tre/) programs for search allowing up to three mismatches between the primers/adapters and the reads to accommodate for sequencing errors. After primers/adapters were removed, the processed reads exhibited multimodal length distribution. The reads from the fungal datasets formed three groups of 184–223 bp, 224–246 bp, and 246–282 bp length. This correlates well with known length variability of ITS sequences from different fungal phyla [89]. Each of the three groups was separately subjected to the OTU clustering and taxonomy assignment procedures, and the results were merged together for further statistical treatment and visualization. ITS1 sequence clustering and taxonomy assignment were performed using USEARCH version 8.1.1756 [90]. For each collection of the related datasets, the OTUs were established by selecting high-quality reads with an expected error rate not exceeding 0.5%. The selected reads were further de-replicated, sorted, clustered at the default 3% difference, and de-chimerized against the UCHIME reference dataset distributed by UNITE [79]. Then, the reads from individual samples were filtered to exclude those with the expected error rate above 6%, and mapped to the OTUs. Taxonomy was assigned using the Warcup training dataset V1 (http://drive5.com/utax/data/utax_warcup_trainset1.tar.gz), with a bootstrap threshold of 50%.

The ITS targeted amplicon sequencing for Flight 3 samples did not yield any product to move forward in generating sequences and this might be due to the low fungal biomass of the samples.

### Statistical analysis

Bar graphs and strip charts of CFU and qPCR data were plotted using Prism (GraphPad Software, version 5.0a; Irvine, CA). Significance (*P* < 0.05) between groups was tested with the Kruskal-Wallis test followed by Dunn’s post-hoc test.

### Amplicon sequence analysis

Bacterial ASV sequences and Fungal OTUs were summarized to the family and/or genus level using QIIME [91]. The ALDEx R package version 2 [70] was used to statistically compare the relative abundances of bacterial family level taxa between the different flights and locations based on the expected values of 128 Dirichlet Monte Carlo instances of centered log ratio (clr) transformed data [71]. A value of zero indicates that organism abundance was equal to the geometric mean abundance. Thus, organisms more abundant than the mean would have positive values, and those less abundant than the mean would have negative values. Significance was based on the Benjamini-Hochberg corrected *P* value of the Kruskal-Wallis statistical test (significance threshold *P* < 0.05). ALDEx2 was also used to compare fungal genus level taxa between flights and differential ASVs and OTUs between samples and controls.

The R script of SourceTracker (version 0.9.1), the contamination predictor tool, was used to assess contamination of the samples [92]. ISS surface wipes were designated as sink and the field and Maxwell negative controls as sources. Samples were rarified to 1000 reads.

QIIME was also used to calculate Shannon’s diversity and taxa richness. Statistical analysis of Shannon’s diversity and taxa richness was performed in Prism using the non-parametric Kruskal-Wallis test with the Benjamini Hochberg FDR multiple test correction.

Genus level counts were clr transformed using the “compositions” package in R [93] and visualized with a heat-map created with the “gplot” package in R. Barplots, boxplots, CCA plots, and pie charts were all created in R.

### Comparison of ISS environmental microbiome with Earth microbiome

The ISS environmental microbiome data were processed by Deblur 1.0.4 [94] trimming at 90 nt with defaults except for setting —min-reads 1 to avoid filtering sequences across samples prior to merging sample sets. The published Earth Microbiome Project 90 nt BIOM table [27] was obtained from ftp://ftp.microbo.me. Deblur 1.0.4 90nt BIOM tables of Hospital Microbiome Project (Qiita study 10,172) and Office Succession Study (Qiita study 10,423) were obtained from Gonzalez et al. [95] using redbiom analysis (https://github.com/biocore/redbiom). Only the reference-hit sOTUs were used across all studies including ISS microbiome datasets. All studies were merged using the BIOM Table Python application programming interface (API). Using the API, sOTUs with fewer than 25 total observed sequences were filtered as was previously performed [96] and samples were rarefied to 1000 sequences per sample. The data were then imported into QIIME2 2018.11 [97] and unique sOTUs were inserted into Greengenes 13_8 [98] using SEPP [99] via the QIIME2 fragment-insertion plugin [100]. For UniFrac, fragment insertion was performed, which was previously shown to ameliorate primer biases [100]. Unweighted UniFrac was computed using Striped UniFrac [101] through QIIME2’s diversity plugin with –p-bypass-tips, principal coordinates were computed using FSVD [102] as used elsewhere [101] and the coordinates were visualized using the EMPeror [103] plugin in QIIME2. Unique sOTUs were assessed in a Jupyter Notebook [104] using the BIOM Table API.

### Controls and nomenclature of the samples

Controls were taken in all steps of the study for all three flight sessions. There was a field control “CTL,” which was a wipe that was opened to the ISS environment but was not used for active sampling and a Maxwell “DNACTL,” which was water that was used during the DNA extraction steps instead of surface or control wipe samples and acted as a DNA extraction reagent control. The field controls were either treated with PMA (“CTL_P”) or left untreated (“CTL”). In total, there were ten controls analyzed during bacterial qPCR and Illumina amplicon sequencing. Likewise, for fungal analysis, the same controls were collected; however, no amplicons were generated for “DNACTL” for either flight nor CTL_P for Flight 1 during qPCR or Illumina library prep and thus were not sent for amplicon sequencing. Similarly, for qPCR and Illumina sequencing, required reagent controls were tested. The samples during this study were designated with flight session number followed by location number (sampling sites). For example, sample number “F1_3” denotes that surface materials were taken from the first flight at location 3 but sample was not treated with PMA, whereas “F1_3P” denotes that same sample was treated with PMA.

## Additional files

Additional file 1: **Figure S1.** Culture- and qPCR-based analyses of microbial burden. **Figure S2.** Assessment of bacterial contamination in the ISS environmental samples. **Figure S3. **Barplot showing the relative abundance of family level bacterial taxa. **Figure S4.** Assessment of microbial alpha diversity. **Figure S5**. Heat map of relative abundances of bacterial genera detected on the ISS across eight locations over a span of 14 months. **Figure S6**. Assessment of fungal contamination in the ISS environmental samples. **Figure S7**. Barplot showing the relative abundance of fungal genera. **Figure S8**. Temporal and spatial distribution of the ISS mycobiome over 2 months and across 8 locations. **Figure S9**. Principal coordinate analysis (PCoA) comparing the viable bacterial population from various regulated indoor environments. **Figure S10**. Spatial and temporal distribution of the bacteria identified on the ISS using culture dependent and independent methods. **Figure S11**. Barplot of metagenomics data showing relative abundances of family level bacterial taxa in each sample. (PDF 2538 kb)

Additional file 2: **Dataset S1**. Amplicon Sequence Variant (ASV) table generated from 16S rRNA gene and fungal ITS region iTag sequencing. (XLSX 182 kb)

Additional file 3: **Table S1.** Summary of results obtained from 16S rRNA gene amplicon sequencing. (DOCX 80 kb)

Additional file 4: **Table S4.** List of unique sequences found on the ISS compared to Earth built environments. (DOCX 103 kb)

Additional file 5: **Table S2.** Information pertaining to the efficiency of the different primers in detecting the most relatively abundant Family level taxa. (DOCX 18 kb)

Additional file 6: **Table S3.** Environmental parameters for ISS samples. (DOCX 21 kb)
