# Supplementary material for: Characterization of the total and viable bacterial and fungal communities associated with the International Space Station surfaces
Source: Microbiome. 2019 Apr 8;7:50. doi: 10.1186/s40168-019-0666-x (PMC6452512; doi:10.1186/s40168-019-0666-x)
Supplement: Supplementary file 1 — Figure S1. Culture- and qPCR-based analyses of microbial burden. Figure S2. Assessment of bacterial contamination in the ISS environmental samples. Figure S3. Barplot showing the relative abundance of family level bacterial taxa. Figure S4. Assessment of microbial alpha diversity. Figure S5. Heat map of relative abundances of bacterial genera detected on the ISS across eight locations over a span of 14 months. Figure S6. Assessment of fungal contamination in the ISS environmental samples. Figure S7. Barplot showing the relative abundance of fungal genera. Figure S8. Temporal and spatial distribution of the ISS mycobiome over 2 months and across 8 locations. Figure S9. Principal coordinate analysis (PCoA) comparing the viable bacterial population from various regulated indoor environments. Figure S10. Spatial and temporal distribution of the bacteria identified on the ISS using culture dependent and independent methods. Figure S11. Barplot of metagenomics data showing relative abundances of family level bacterial taxa in each sample. (PDF 2538 kb) [file 40168_2019_666_MOESM1_ESM.pdf]

Supplemental Figure Captions

Fig. S1 Culture- and qPCR-based analyses of microbial burden.

A total of 133 bacterial isolates (A) and 81 fungal isolates (B) that were cultured from eight locations during three flights, were picked for identification. The bar length (middle panel) represents the number of isolates that were identified as a particular species or genus (left panel). The bar color indicates the same genus. The color filled checkerboard (right panel) indicates from which samples the bacteria were cultured from with black representing flight 1, red, flight 2 and black, flight 3. (C) Percent of intact/viable bacteria and fungi detected on the ISS that were able to be cultured. The number of culturable bacteria or fungi, determined by CFU/m^2, was divided by the total number of intact/viable bacteria or fungi (both culturable and non culturable) as determined by PMA- qPCR. The bar graph represents the percent of intact/viable bacteria or fungi that were able to be cultured from the ISS (n=24 wipes) using standard laboratory techniques. (D) Comparison of PMA (intact/viable) and non-PMA (dead and cells with a compromised cell membrane) treated samples analyzed with qPCR. The bar graph represents the gene copy number/m^2 for bacteria (16S rRNA gene) or fungi (ITS region) in either PMA or non-PMA treated samples. The height of the bar represents the average gene copy number/m^2 for 24 samples. The whiskers pinpoint the lowest and highest value in that group. No statistically significant differences were observed between PMA vs non-PMA treated samples for both bacteria and fungi. Kruskal-Wallis test followed by Dunn's post hoc test $P > 0.05$. (E**)** Percent of viable bacterial (left) and fungal (right graph) population as measured by number of16S rRNA gene and ITS region copies via qPCR. Sample location numbers are shown in X-axis (L1 to L8). The percentages of viable bacterial or fungal population calculated by dividing 16S rRNA gene

copies or ITS region of PMA (intact/viable) by non-PMA (dead and intact/viable) treated samples are shown in Y-axis.

Fig. S2 Assessment of bacterial contamination in the ISS environmental samples.

(A) Bar graph showing the total read count for each sample and each control. “CTL” indicates the sampling wipe that was exposed to the ISS environment but did not touch any surfaces and “DNACTL” was water added to the negative control for extraction instead of material obtained from the surface/control wipe. (B) SourceTracker was used to examine if and in what proportion the ISS surface bacterial microbiome was associated with contaminating bacterial DNA from the sampling wipes or contaminated with DNA during processing, extraction and sequencing. This was done by comparing the ASV sequences found in the controls (“source”) with that present in the samples (“sink”). Each pie graph represents one sample. The blue represents the ASVs unique to the ISS surface samples. The pink, beige and purple slices represent the proportion of ASVs in the ISS surface samples that were similar to those found in 3 controls from F1 (pink), 3 from F2 (beige) and 4 from F3 (purple). The number after F1, F2, F3 refers to the location sampled and “P” indicates a sample that was treated with PMA before DNA extraction. Read counts were rarified to 1000 reads.

Fig. S3 Barplot showing the relative abundance of family level bacterial taxa.

Each bar represents a sample collected from particular location and during a specific Flight sampling session. F1, F2, F3 refers to one of the three flight sampling sessions, with the number after the underscore indicating the location it was sampled from (i.e. F1_1= flight 1, location 1).. The colors in each bar represent a taxon and the height of that colored box represents the relative proportion of that taxon within a sample. The remaining fraction, colored grey, groups taxa that were less than 2% abundance in each sample. The legend is read from bottom to top, with the

bottom taxon on the legend corresponding to the bottom colored on the barplot. (A) untreated samples and (B) PMA treated samples.

Fig. S4 Assessment of microbial alpha diversity.

Bacterial diversity within a sample (based on sequences that were summarized down to the Family level) was measured with (A) Shannon's diversity index, which measures organism presence/absence and its relative abundance within a sample and (B) Taxa richness, which reports the number of unique taxa within a particular sample. (A) Each symbol on the graph represents one location sampled (1-8) with the line representing the median for all samples. The higher the index the greater the bacterial diversity found within a sample. Since Shannon's diversity index is a logarithmic number with base 2, a value of "4" is 2x higher than a value of "3". The average index for F2 samples was statistically significantly higher than that of F1 and F3 samples (P <0.05). There were no differences (P >0.05) in the diversity index between PMA and non-PMA treated samples for each flight. (B) Each point on the graph represents a location with the line representing the median for all samples. F2 samples were statistically significantly richer than F3 samples but not F1 samples. There were no differences in richness between PMA and non-PMA treated samples at each Flight. Fungal alpha diversity was also measured using (C) Shannon's diversity index and (D) Taxa richness. There were no statistically significant differences (P > 0.05) in Shannon's diversity index nor taxa richness between F1 and F2 samples and between PMA and non-PMA treated samples at each Flight. The Kruskal-Wallis test with the Benjamini Hochberg FDR multiple test correction was used for both bacterial and fungal statistical analyses.

Fig. S5 Heat map of relative abundances of bacterial genera detected on the ISS across eight locations over a span of 14 months.

24 surface wipes, were collected from 8 locations over 3 flight sampling sessions, spanning 14 months. DNA was sent for 16S rRNA gene targeted sequencing and the sequences taxonomically assigned using the SILVA database. The ASV table was summarized to the genus level and read counts were transformed using centered log ratios. With centered log ratio data, zero represents the geometric mean abundance (i.e. the average relative abundance of all organisms within a sample), thus the higher a value is above zero the more abundant that organism is compared to all others in that sample. The highest relative abundance is depicted in red and the lowest in light yellow. Grey represents those taxa that had raw read counts of zero. F1, F2, F3 refers to one of the three flight sampling sessions, with the number after the underscore indicating the location it was sampled from (i.e. F1_1= flight 1, location 1). "P" at the end indicates that the sample was treated with PMA before DNA extraction. Out of 121 taxa identified, 77 could be assigned to the genus level. The genera were classified based on whether published studies have found them to form spores and/or biofilms and whether they are associated with the environment or humans. The pie chart represents the percent of each classification detected in the dataset

Fig. S6 Assessment of fungal contamination in the ISS environmental samples.

SourceTracker was used to examine if and in what proportion the ISS fungal surface microbiome was associated with contaminating fungal DNA from the sampling wipes or contaminated with DNA during processing, DNA extraction and sequencing. This was done by comparing the OTUs sequences found in the controls ("source") with that present in the samples ("sink"). Each pie graph represents one sample. The beige represents the OTUs unique to the ISS samples and the pink represents the proportion of OTUs in the ISS samples that were similar to those found in

three controls from F1 and F2. Read counts were rarified to 1000 reads. The number after F1 and F2 refer to the location sampled and "P" indicates a sample that was treated with PMA before DNA extraction. (B) Bar graph showing the total read count for each sample and each control. "CTL" indicates the sampling wipe that was exposed to the ISS environment but did not touch any surfaces. NB: A DNA control "DNACTL" (water added for extraction instead of material obtained from the surface/control wipe) and a F1 CTL_P sample were processed however they did not produce any amplicons and thus was not sent for sequencing.

Fig. S7 Barplot showing the relative abundance of fungal genera.

Barplot showing the relative abundances of fungi, summarized to the genus level, of 16 wipes collected during Flight 1 and Flight 2, at 8 different locations across the ISS. Each bar represents a sample collected from particular location and during a specific Flight sampling session. The letter "P" after the location number signifies PMA treatment of the sample (i.e F1_1P means sample collected from Flight 1, location 1, PMA treatment whereas F2_5 means sample collected from Flight 2, location 5, not treated with PMA). The colors in each bar represent a genus taxon and the height of that colored box represents the relative proportion of that genus within a sample. The legend is read from bottom to top, with the bottom taxon on the legend corresponding to the bottom colored on the barplot. ALDEx2 did not report any statistically significant differences in the mycobiome between F1 and F2.

Fig. S8 Temporal and spatial distribution of the ISS mycobiome over 2 months and across 8 locations.

Boxplots show the temporal (A) and spatial (B) distribution of the most relatively abundant fungal genera in the 16 samples. (A) The box in each graph signifies the 75% (upper) and 25% (lower) quartiles and thus shows the percent abundances for 50% of the samples (N=8). The

black line inside the box represents the median. The bottom whisker represents the lowest datum still within the 1.5 interquartile range (IQR) of the lower quartile, with the top whisker representing the highest datum still within the 1.5 IQR of the upper quartile. Open circle are outliers. "F" indicates Flight, with the "P" after the number signifying PMA treatment. Significance was measured using ALDEx2 and based on the Benjamini-Hochberg corrected P value of the Kruskal-Wallis test (significance threshold, $P<0.05$). (B) Since there are only 2 samples, the top of the box represents one value and the bottom the second value, with the line representing the median. "L" indicates Location with the "P" after the number signifying PMA treatment. No statistical test was performed as there were only 2 values.

Fig. S9 Principal coordinate analysis (PCoA) comparing the viable bacterial population from various regulated indoor environments.

PCoA plots of Bray-Curtis dissimilarity were used to compare PMA treated samples (i.e. intact cell membrane/viable) from other "harsh" indoor environments that have been collected by our group. Samples include (i) dust collected from two cleanrooms at JPL; the spacecraft assembly facility (SAF) and Bldg 103 [2], (ii) dust collected from vacuum bags and HEPA filters from the ISS [2] and (iii) surface samples collected from the inflated Lunar/Mars analogue habitat (ILMAH) [107]. The samples from this study are labelled as ISS.Surface_Fx.PMA, with x denoting collective samples from either F1, F2 or F3. Points closer together are more similar in the proportions and type of bacteria present than those further away on the plot. Points were plotted on this 3-axis plane representing 69% of the observed variation amongst all samples.

Fig. S10 Spatial and temporal distribution of the bacteria identified on the ISS using culture dependent and independent methods.

This chart summarizes which cultured bacteria were also detected with 16S rRNA sequencing. The far left column lists all bacteria that were cultured, summarized to the genus level. A black box indicates that the genus was cultured from a sample (i.e. *Acinetobacter* was cultured from the sample collected from location "1", flight 2 "ISS2: for *Acinetobacter*). A green box indicates that the genus was detected from a sample treated with PMA (i.e. intact, potentially viable) and a red box indicates that the genus was detected from a sample that was not treated with PMA (i.e. total).

Fig. S11 Barplot of metagenomics data showing relative abundances of family level bacterial taxa in each sample.

Each bar represents a sample collected from particular location and during a specific Flight sampling session. The colors in each bar represent a taxon and the height of that colored box represents the relative proportion of that taxon within a sample. The remaining fraction, colored grey, groups taxa that were less than 2% abundance in each sample. The legend is read from bottom to top, with the bottom taxon on the legend corresponding to the bottom colored on the barplot. The samples shown in this graph are non-PMA treated. NB: The colors associated with each taxon in this figure match the colors for each taxon shown in the microbiome barplot in Fig. S3.

Fig. S1

A

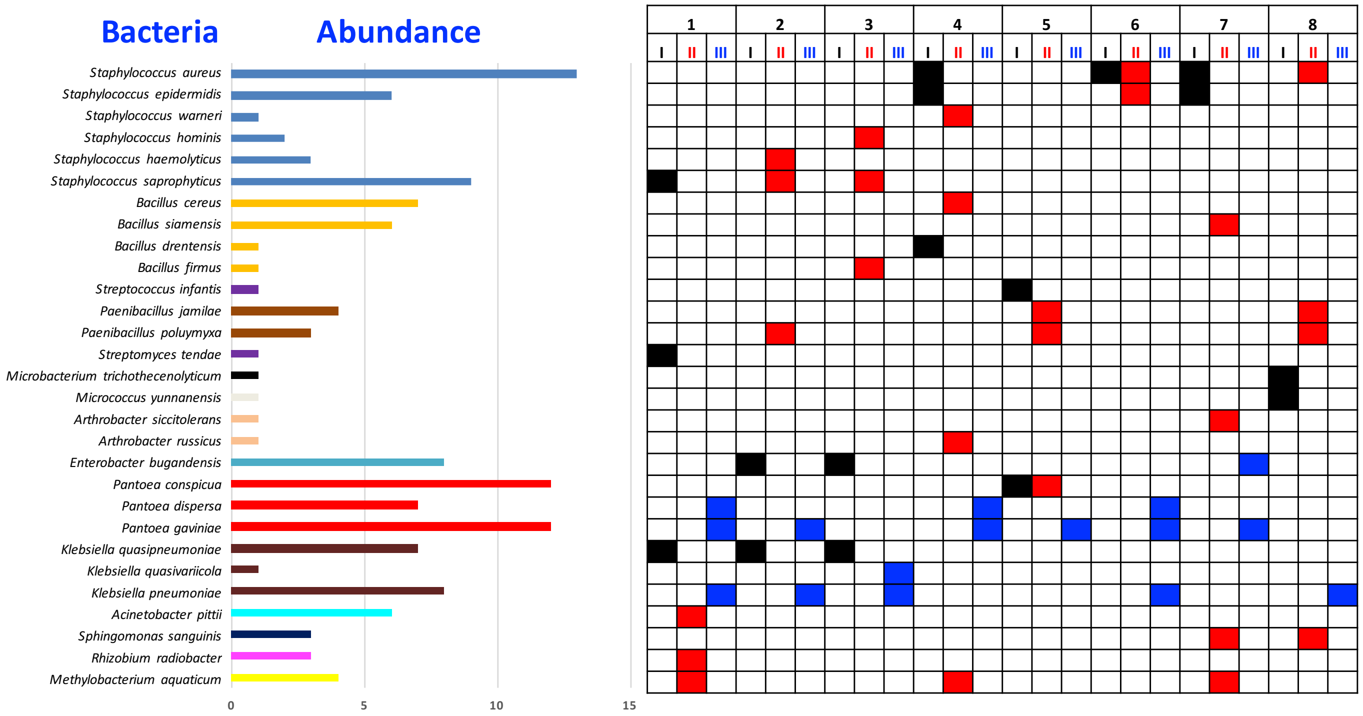

Bacteria
Abundance
Staphylococcus aureus
Staphylococcus epidermidis
Staphylococcus warneri
Staphylococcus hominis
Staphylococcus haemolyticus
Staphylococcus saprophyticus
Bacillus cereus
Bacillus siamensis
Bacillus drentensis
Bacillus firmus
Streptococcus infantis
Paenibacillus jamilae
Paenibacillus poluymyxa
Streptomyces tendae
Microbacterium trichothecenolyticum
Micrococcus yunnanensis
Arthrobacter siccitolerans
Arthrobacter russicus
Enterobacter bugandensis
Pantoea conspicua
Pantoea dispersa
Pantoea gaviniae
Klebsiella quasipneumoniae
Klebsiella quasivariicola
Klebsiella pneumoniae
Acinetobacter pittii
Sphingomonas sanguinis
Rhizobium radiobacter
Methylobacterium aquaticum
0
5
10
15
1
2
3
4
5
6
7
8
I II III

B

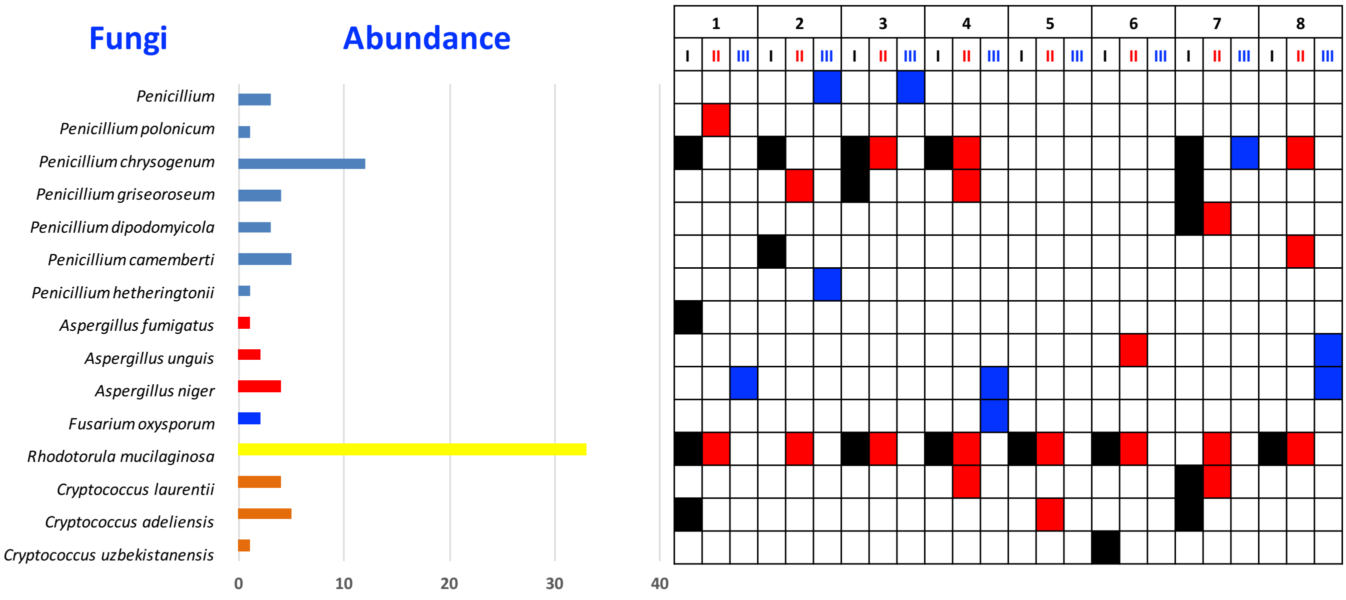

Fungi
Abundance
Penicillium
Penicillium polonicum
Penicillium chrysogenum
Penicillium griseoroseum
Penicillium dipodomyicola
Penicillium camemberti
Penicillium hetheringtonii
Aspergillus fumigatus
Aspergillus unguis
Aspergillus niger
Fusarium oxysporum
Rhodotorula mucilaginosa
Cryptococcus laurentii
Cryptococcus adeliensis
Cryptococcus uzbekistanensis
0
10
20
30
40
1
2
3
4
5
6
7
8
I II III

Fig. S1

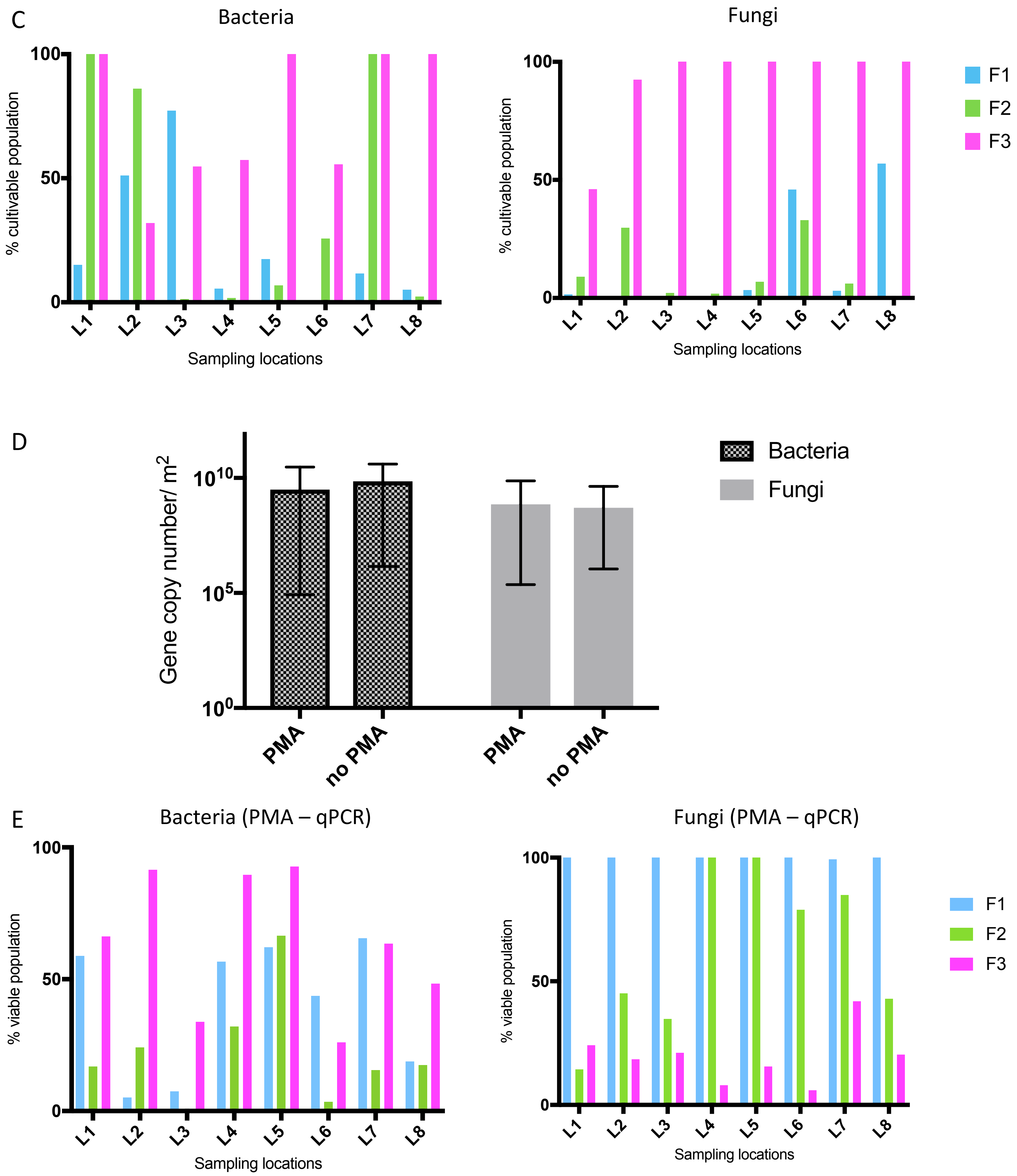
C
Bacteria
Fungi
100
50
0
% cultivable population
L1
L2
L3
L4
L5
L6
L7
L8
Sampling locations
F1
F2
F3
D
Gene copy number/ m2
10^10
10^5
10^0
PMA
no PMA
PMA
no PMA
Bacteria
Fungi
E
Bacteria (PMA – qPCR)
Fungi (PMA – qPCR)
% viable population
Sampling locations

Fig. S2A

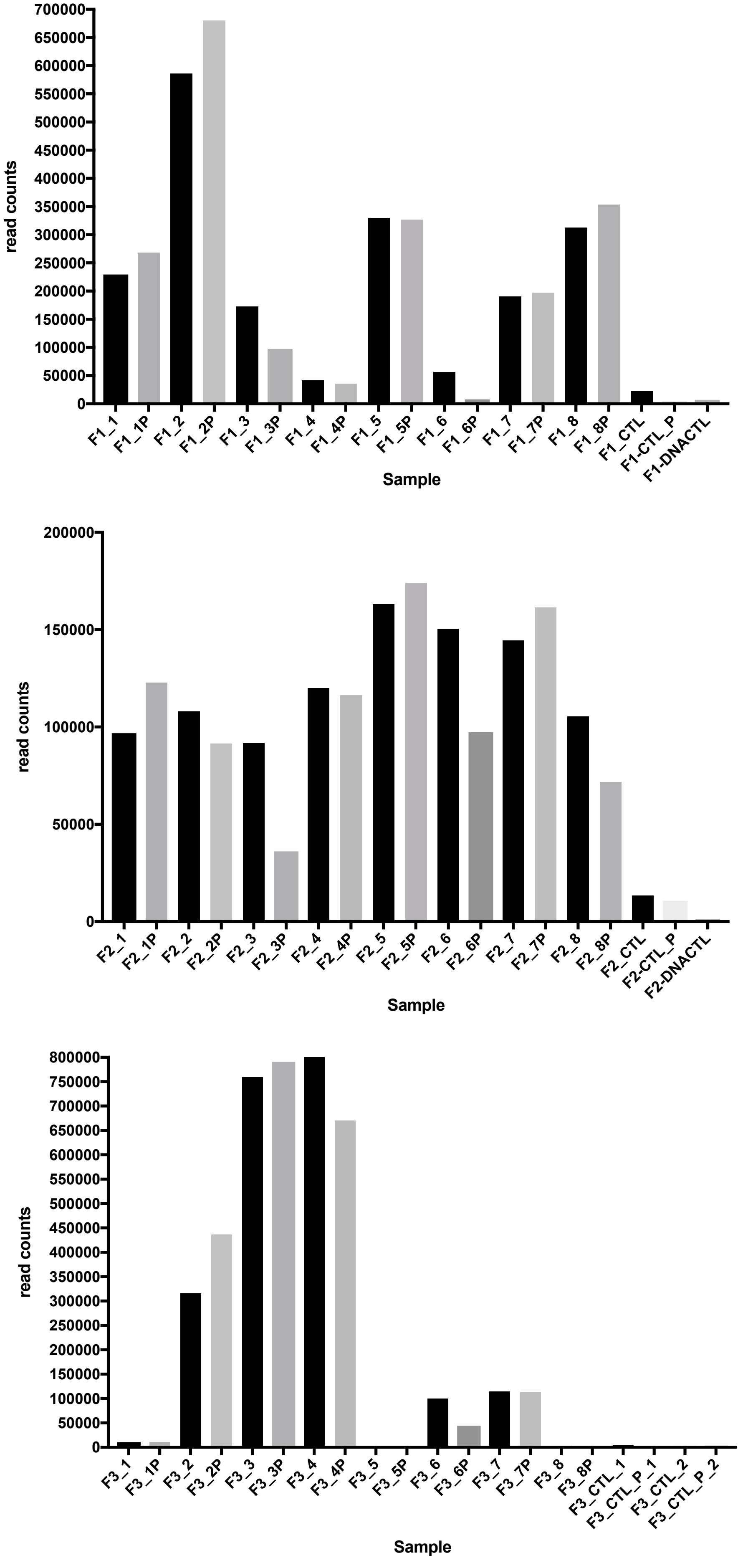

F1

F2

F3

Fig. S2B

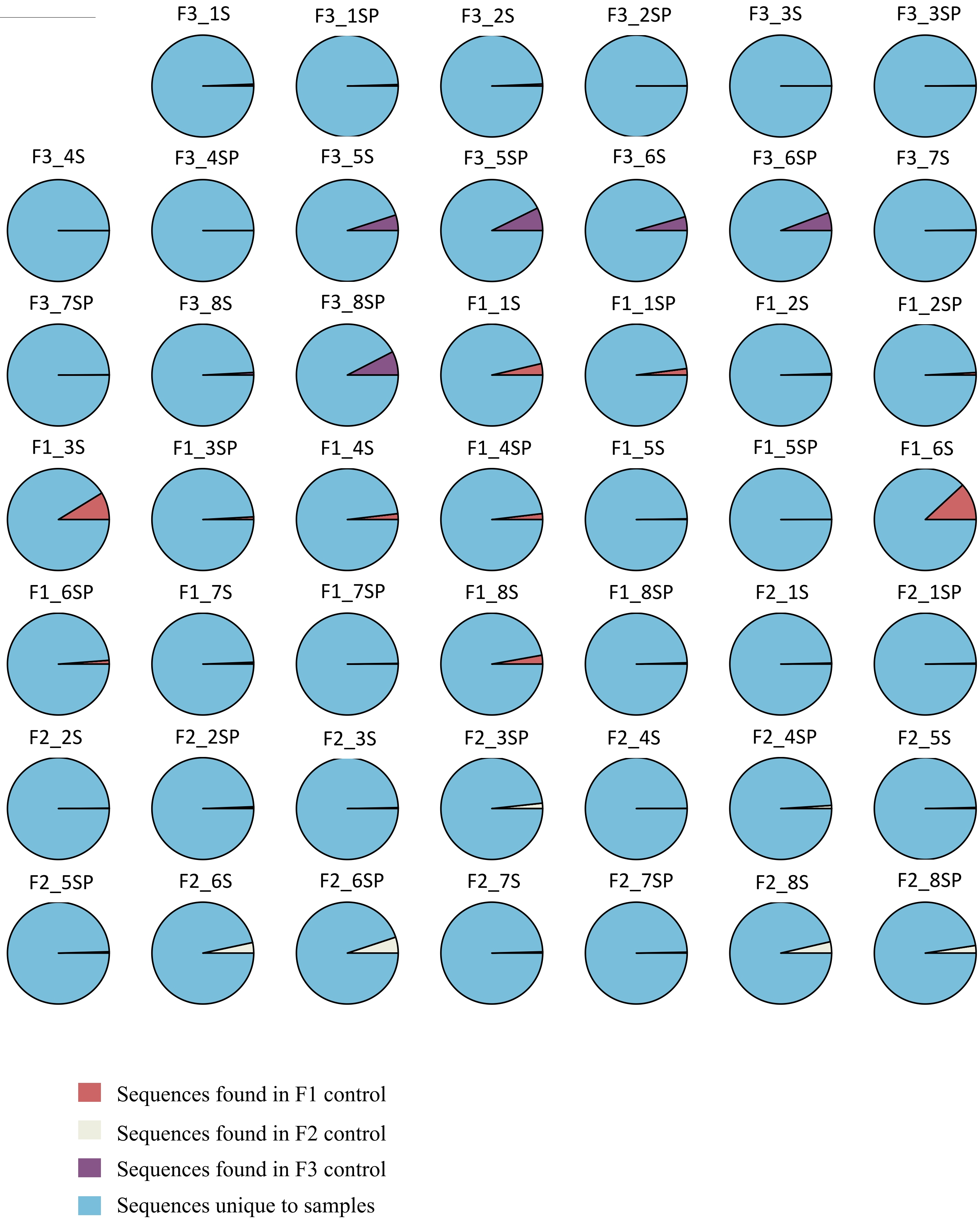
F3_1S
F3_1SP
F3_2S
F3_2SP
F3_3S
F3_3SP
F3_4S
F3_4SP
F3_5S
F3_5SP
F3_6S
F3_6SP
F3_7S
F3_7SP
F3_8S
F3_8SP
F1_1S
F1_1SP
F1_2S
F1_2SP
F1_3S
F1_3SP
F1_4S
F1_4SP
F1_5S
F1_5SP
F1_6S
F1_6SP
F1_7S
F1_7SP
F1_8S
F1_8SP
F2_1S
F2_1SP
F2_2S
F2_2SP
F2_3S
F2_3SP
F2_4S
F2_4SP
F2_5S
F2_5SP
F2_6S
F2_6SP
F2_7S
F2_7SP
F2_8S
F2_8SP
Sequences found in F1 control
Sequences found in F2 control
Sequences found in F3 control
Sequences unique to samples

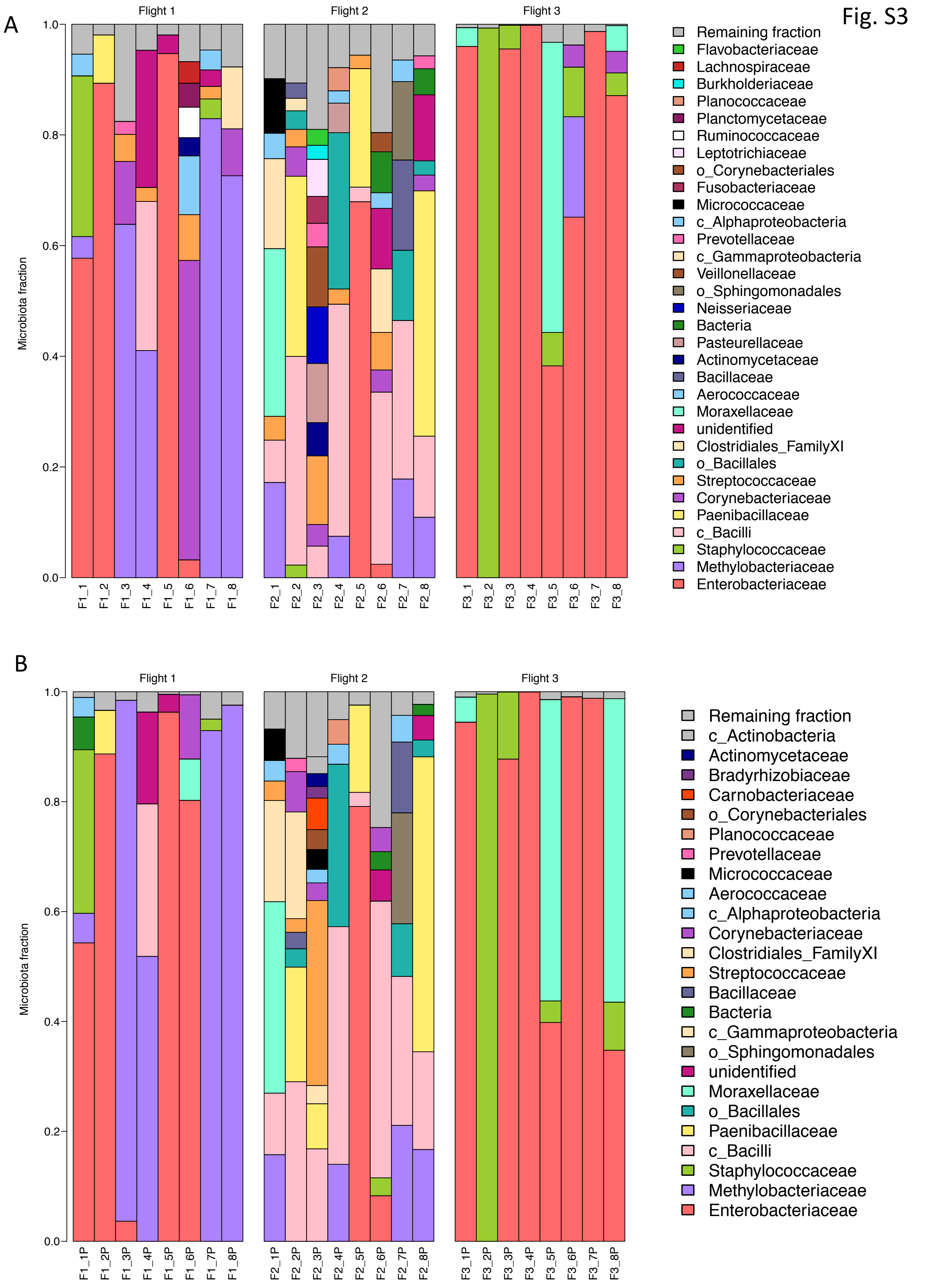
Fig. S3
A
Flight 1
Flight 2
Flight 3
Microbiota fraction
0.0
0.2
0.4
0.6
0.8
1.0
F1_1
F1_2
F1_3
F1_4
F1_5
F1_6
F1_7
F1_8
F2_1
F2_2
F2_3
F2_4
F2_5
F2_6
F2_7
F2_8
F3_1
F3_2
F3_3
F3_4
F3_5
F3_6
F3_7
F3_8
Remaining fraction
Flavobacteriaceae
Lachnospiraceae
Burkholderiaceae
Planococcaceae
Planctomycetaceae
Ruminococcaceae
Leptotrichiaceae
o_Corynebacteriales
Fusobacteriaceae
Micrococcaceae
c_Alphaproteobacteria
Prevotellaceae
c_Gammaproteobacteria
Veillonellaceae
o_Sphingomonadales
Neisseriaceae
Bacteria
Pasteurellaceae
Actinomycetaceae
Bacillaceae
Aerococcaceae
Moraxellaceae
unidentified
Clostridiales_FamilyXI
o_Bacillales
Streptococcaceae
Corynebacteriaceae
Paenibacillaceae
c_Bacilli
Staphylococcaceae
Methylobacteriaceae
Enterobacteriaceae
B
Flight 1
Flight 2
Flight 3
Microbiota fraction
0.0
0.2
0.4
0.6
0.8
1.0
F1_1P
F1_2P
F1_3P
F1_4P
F1_5P
F1_6P
F1_7P
F1_8P
F2_1P
F2_2P
F2_3P
F2_4P
F2_5P
F2_6P
F2_7P
F2_8P
F3_1P
F3_2P
F3_3P
F3_4P
F3_5P
F3_6P
F3_7P
F3_8P
Remaining fraction
c_Actinobacteria
Actinomycetaceae
Bradyrhizobiaceae
Carnobacteriaceae
o_Corynebacteriales
Planococcaceae
Prevotellaceae
Micrococcaceae
Aerococcaceae
c_Alphaproteobacteria
Corynebacteriaceae
Clostridiales_FamilyXI
Streptococcaceae
Bacillaceae
Bacteria
c_Gammaproteobacteria
o_Sphingomonadales
unidentified
Moraxellaceae
o_Bacillales
Paenibacillaceae
c_Bacilli
Staphylococcaceae
Methylobacteriaceae
Enterobacteriaceae

Fig. S4

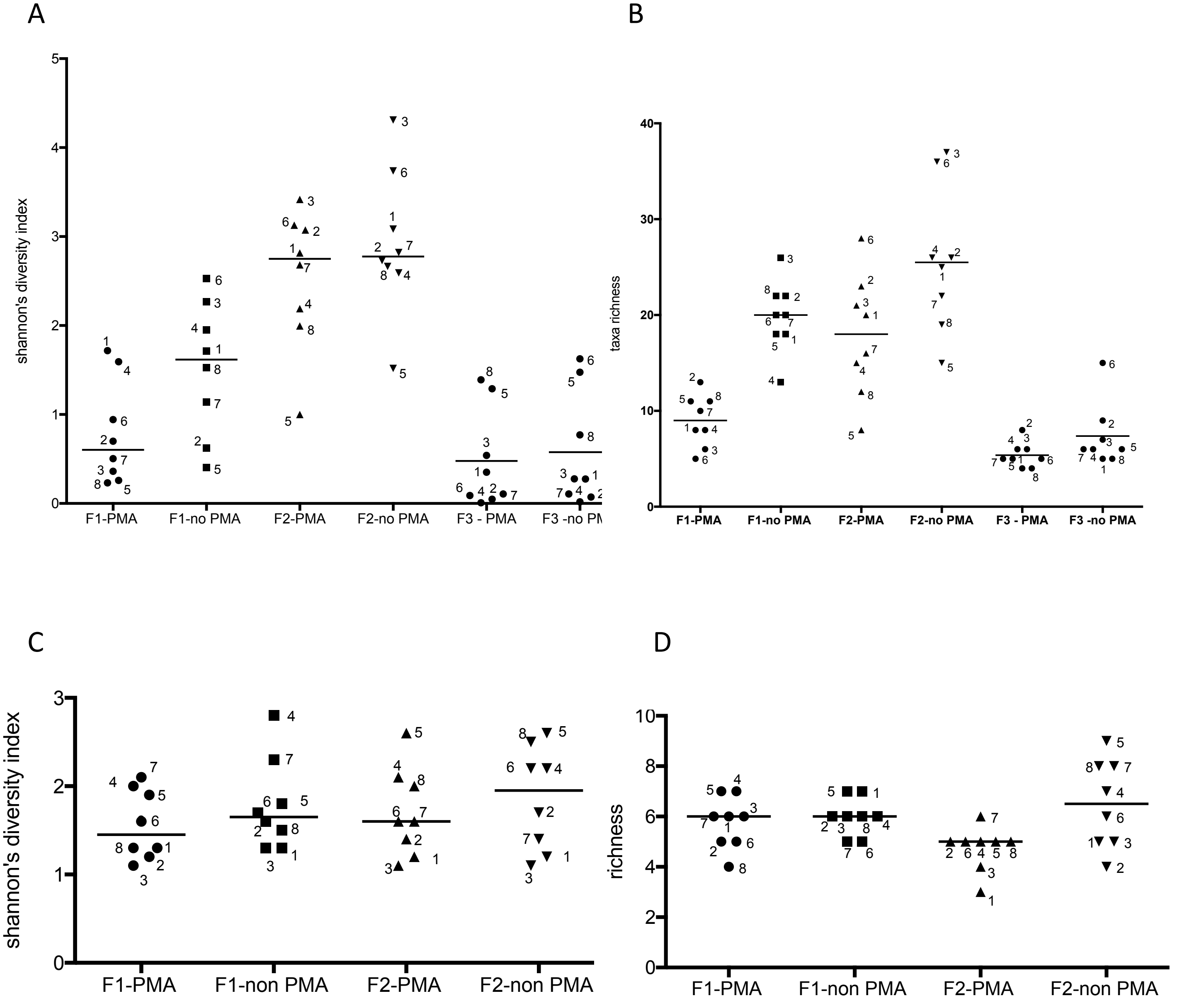
A
shannon's diversity index
F1-PMA
F1-no PMA
F2-PMA
F2-no PMA
F3 - PMA
F3 -no PM
B
taxa richness
F1-PMA
F1-no PMA
F2-PMA
F2-no PMA
F3 - PMA
F3 -no PMA
C
shannon's diversity index
F1-PMA
F1-non PMA
F2-PMA
F2-non PMA
D
richness
F1-PMA
F1-non PMA
F2-PMA
F2-non PMA

Fig. S5

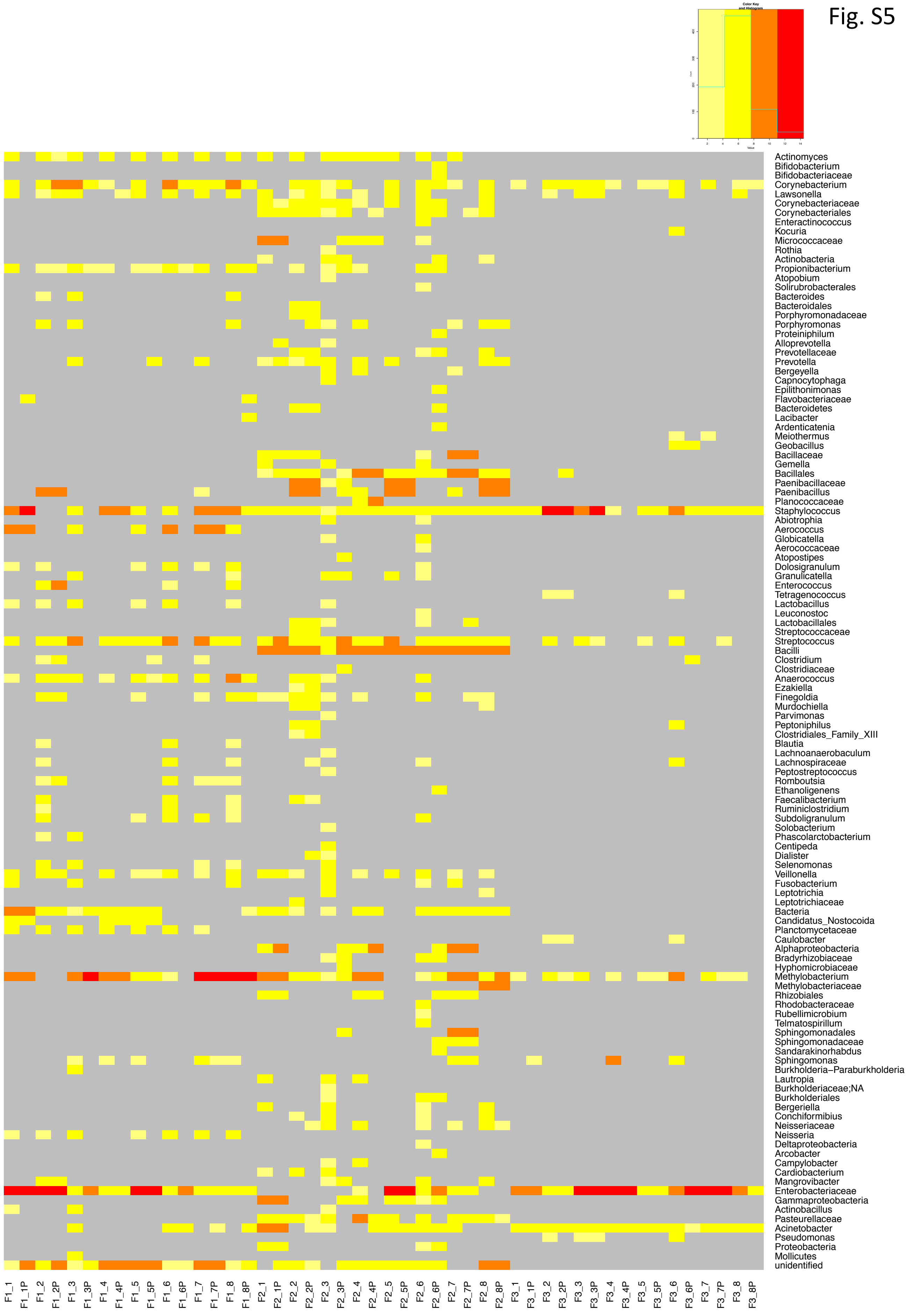
Color Key and Histogram
Value
Count
Actinomyces
Bifidobacterium
Bifidobacteriaceae
Corynebacterium
Lawsonella
Corynebacteriaceae
Corynebacteriales
Enteractinococcus
Kocuria
Micrococcaceae
Rothia
Actinobacteria
Propionibacterium
Atopobium
Solirubrobacterales
Bacteroides
Bacteroidales
Porphyromonadaceae
Porphyromonas
Proteiniphilum
Alloprevotella
Prevotellaceae
Prevotella
Bergeyella
Capnocytophaga
Epilithonimonas
Flavobacteriaceae
Bacteroidetes
Lacibacter
Ardenticatenia
Meiothermus
Geobacillus
Bacillaceae
Gemella
Bacillales
Paenibacillaceae
Paenibacillus
Planococcaceae
Staphylococcus
Abiotrophia
Aerococcus
Globicatella
Aerococcaceae
Atopostipes
Dolosigranulum
Granulicatella
Enterococcus
Tetragenococcus
Lactobacillus
Leuconostoc
Lactobacillales
Streptococcaceae
Streptococcus
Bacilli
Clostridium
Clostridiaceae
Anaerococcus
Ezakiella
Finegoldia
Murdochiella
Parvimonas
Peptoniphilus
Clostridiales_Family_XIII
Blautia
Lachnoanaerobaculum
Lachnospiraceae
Peptostreptococcus
Romboutsia
Ethanoligenens
Faecalibacterium
Ruminiclostridium
Subdoligranulum
Solobacterium
Phascolarctobacterium
Centipeda
Dialister
Selenomonas
Veillonella
Fusobacterium
Leptotrichia
Leptotrichiaceae
Bacteria
Candidatus_Nostocoida
Planctomycetaceae
Caulobacter
Alphaproteobacteria
Bradyrhizobiaceae
Hyphomicrobiaceae
Methylobacterium
Methylobacteriaceae
Rhizobiales
Rhodobacteraceae
Rubellimicrobium
Telmatospirillum
Sphingomonadales
Sphingomonadaceae
Sandarakinorhabdus
Sphingomonas
Burkholderia−Paraburkholderia
Lautropia
Burkholderiaceae;NA
Burkholderiales
Bergeriella
Conchiformibius
Neisseriaceae
Neisseria
Deltaproteobacteria
Arcobacter
Campylobacter
Cardiobacterium
Mangrovibacter
Enterobacteriaceae
Gammaproteobacteria
Actinobacillus
Pasteurellaceae
Acinetobacter
Pseudomonas
Proteobacteria
Mollicutes
unidentified
F1_1
F1_1P
F1_2
F1_2P
F1_3
F1_3P
F1_4
F1_4P
F1_5
F1_5P
F1_6
F1_6P
F1_7
F1_7P
F1_8
F1_8P
F2_1
F2_1P
F2_2
F2_2P
F2_3
F2_3P
F2_4
F2_4P
F2_5
F2_5P
F2_6
F2_6P
F2_7
F2_7P
F2_8
F2_8P
F3_1
F3_1P
F3_2
F3_2P
F3_3
F3_3P
F3_4
F3_4P
F3_5
F3_5P
F3_6
F3_6P
F3_7
F3_7P
F3_8
F3_8P

Fig. S6A

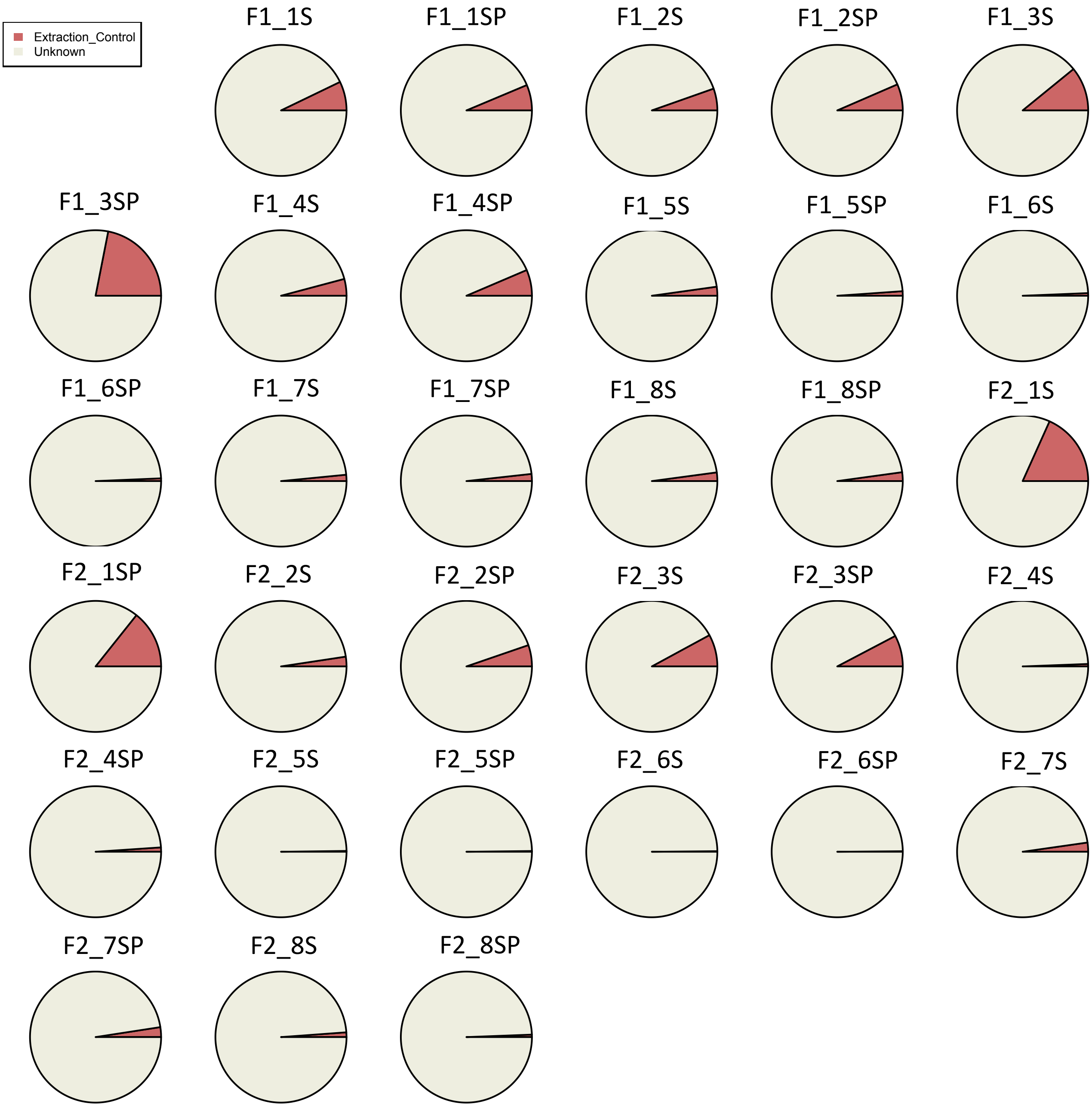
Extraction_Control
Unknown
F1_1S
F1_1SP
F1_2S
F1_2SP
F1_3S
F1_3SP
F1_4S
F1_4SP
F1_5S
F1_5SP
F1_6S
F1_6SP
F1_7S
F1_7SP
F1_8S
F1_8SP
F2_1S
F2_1SP
F2_2S
F2_2SP
F2_3S
F2_3SP
F2_4S
F2_4SP
F2_5S
F2_5SP
F2_6S
F2_6SP
F2_7S
F2_7SP
F2_8S
F2_8SP

Fig. S6B

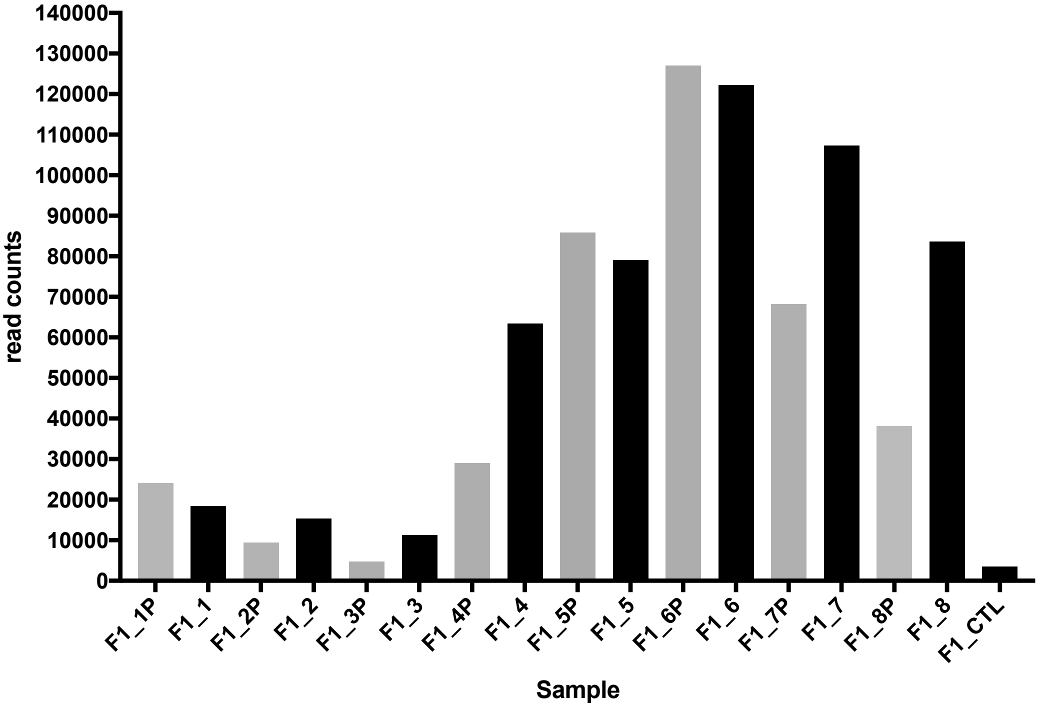

140000
130000
120000
110000
100000
90000
80000
70000
60000
50000
40000
30000
20000
10000
0
read counts
F1_1P
F1_1
F1_2P
F1_2
F1_3P
F1_3
F1_4P
F1_4
F1_5P
F1_5
F1_6P
F1_6
F1_7P
F1_7
F1_8P
F1_8
F1_CTL
Sample

F1

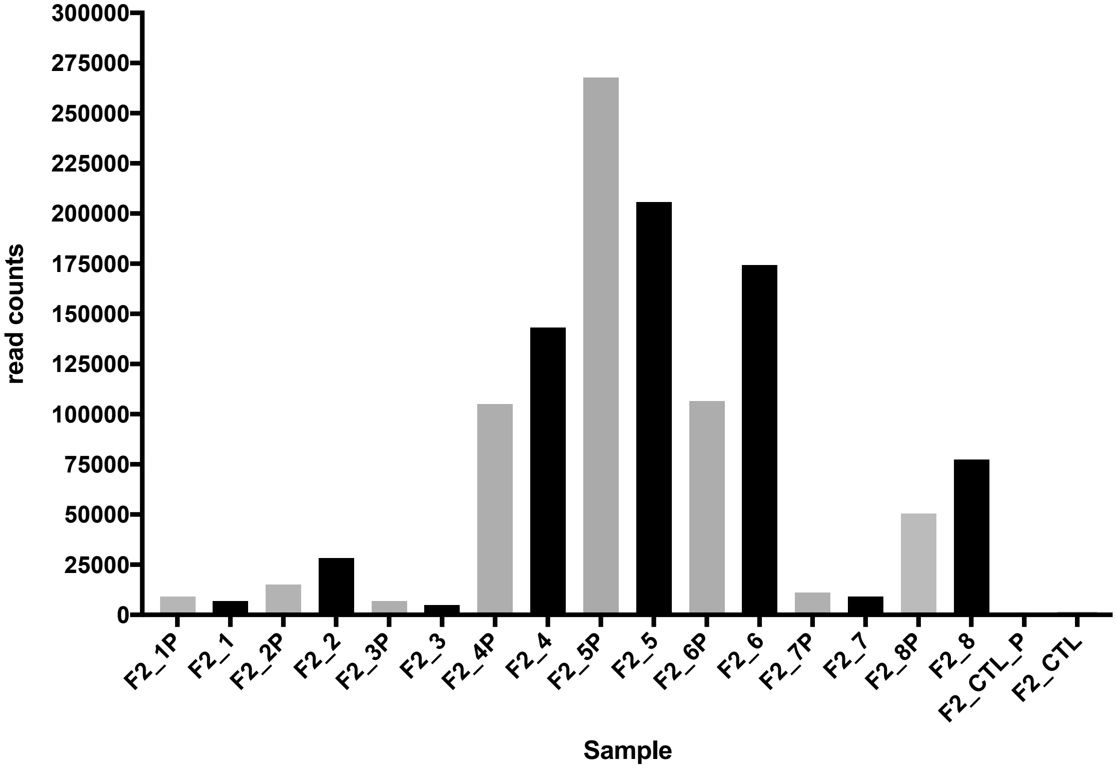

300000
275000
250000
225000
200000
175000
150000
125000
100000
75000
50000
25000
0
read counts
F2_1P
F2_1
F2_2P
F2_2
F2_3P
F2_3
F2_4P
F2_4
F2_5P
F2_5
F2_6P
F2_6
F2_7P
F2_7
F2_8P
F2_8
F2_CTL_P
F2_CTL
Sample

F2

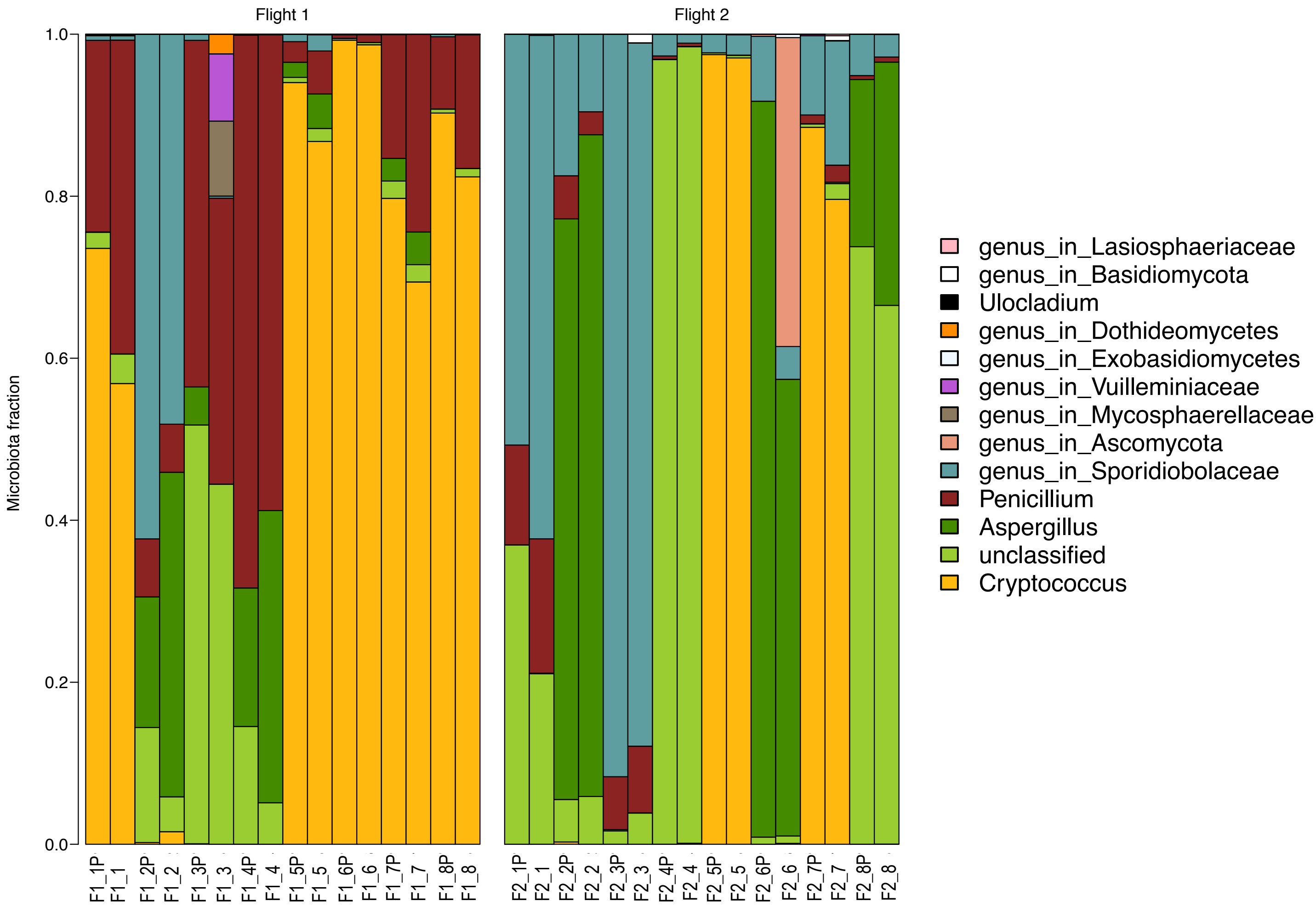
Flight 1
Flight 2
Microbiota fraction
0.0
0.2
0.4
0.6
0.8
1.0
F1_1P
F1_1
F1_2P
F1_2
F1_3P
F1_3
F1_4P
F1_4
F1_5P
F1_5
F1_6P
F1_6
F1_7P
F1_7
F1_8P
F1_8
F2_1P
F2_1
F2_2P
F2_2
F2_3P
F2_3
F2_4P
F2_4
F2_5P
F2_5
F2_6P
F2_6
F2_7P
F2_7
F2_8P
F2_8
genus_in_Lasiosphaeriaceae
genus_in_Basidiomycota
Ulocladium
genus_in_Dothideomycetes
genus_in_Exobasidiomycetes
genus_in_Vuilleminiaceae
genus_in_Mycosphaerellaceae
genus_in_Ascomycota
genus_in_Sporidiobolaceae
Penicillium
Aspergillus
unclassified
Cryptococcus

Fig. S8

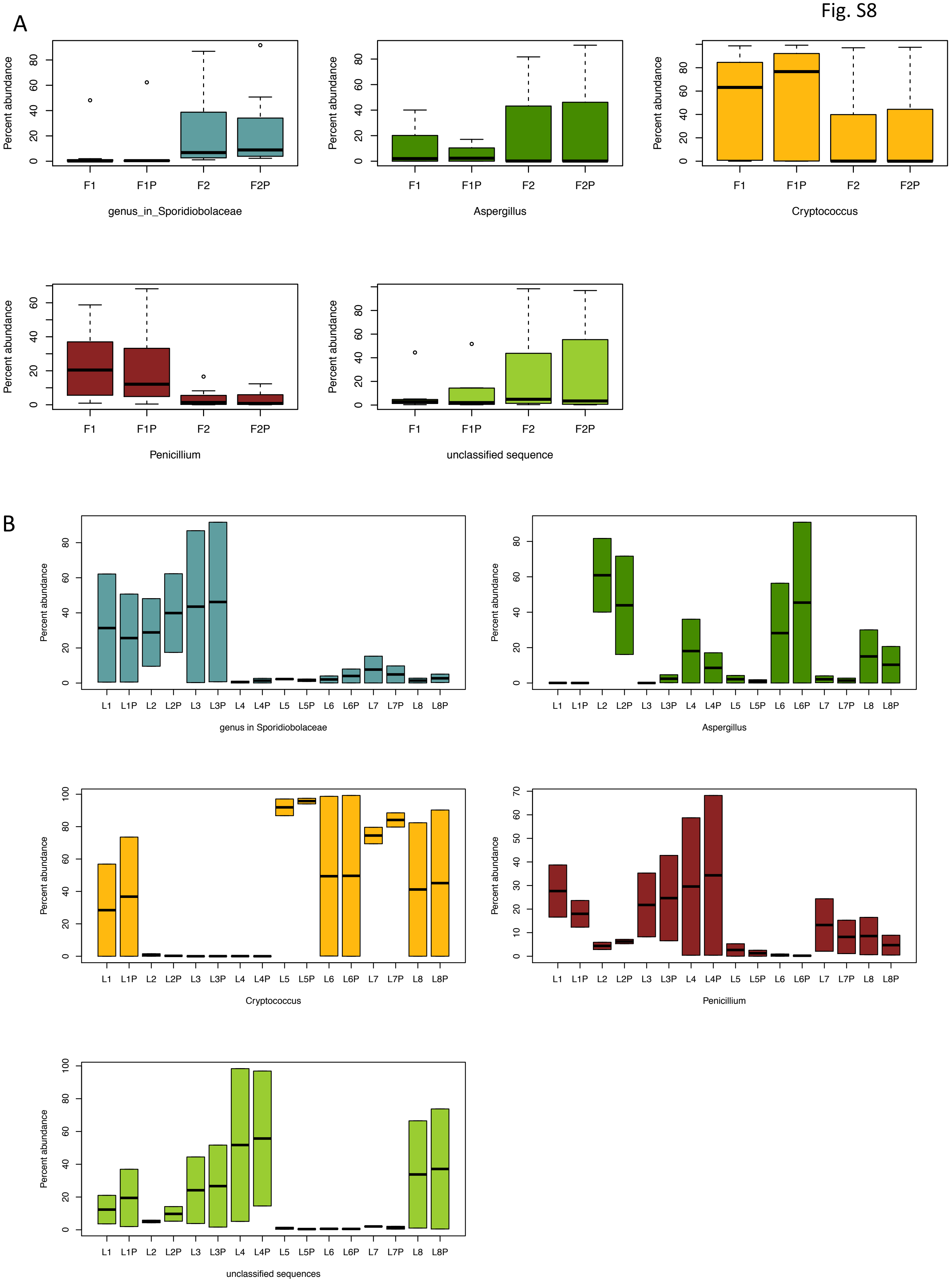

A
Percent abundance
F1
F1P
F2
F2P
genus_in_Sporidiobolaceae
Aspergillus
Cryptococcus
Penicillium
unclassified sequence
B
L1 L1P L2 L2P L3 L3P L4 L4P L5 L5P L6 L6P L7 L7P L8 L8P
genus in Sporidiobolaceae
Aspergillus
Cryptococcus
Penicillium
unclassified sequences

Fig. S9

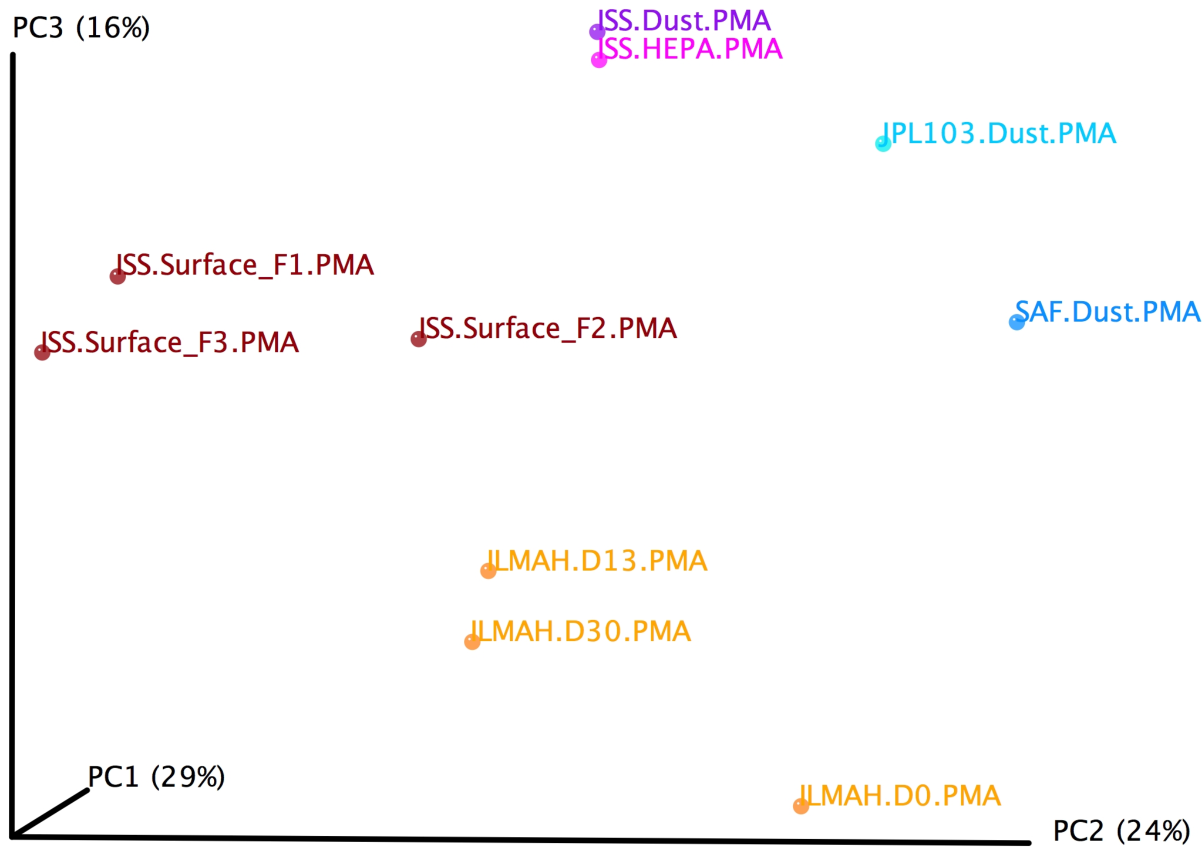

PC3 (16%)
ISS.Dust.PMA
ISS.HEPA.PMA
JPL103.Dust.PMA
ISS.Surface_F1.PMA
ISS.Surface_F3.PMA
ISS.Surface_F2.PMA
SAF.Dust.PMA
ILMAH.D13.PMA
ILMAH.D30.PMA
PC1 (29%)
ILMAH.D0.PMA
PC2 (24%)

Fig. S10

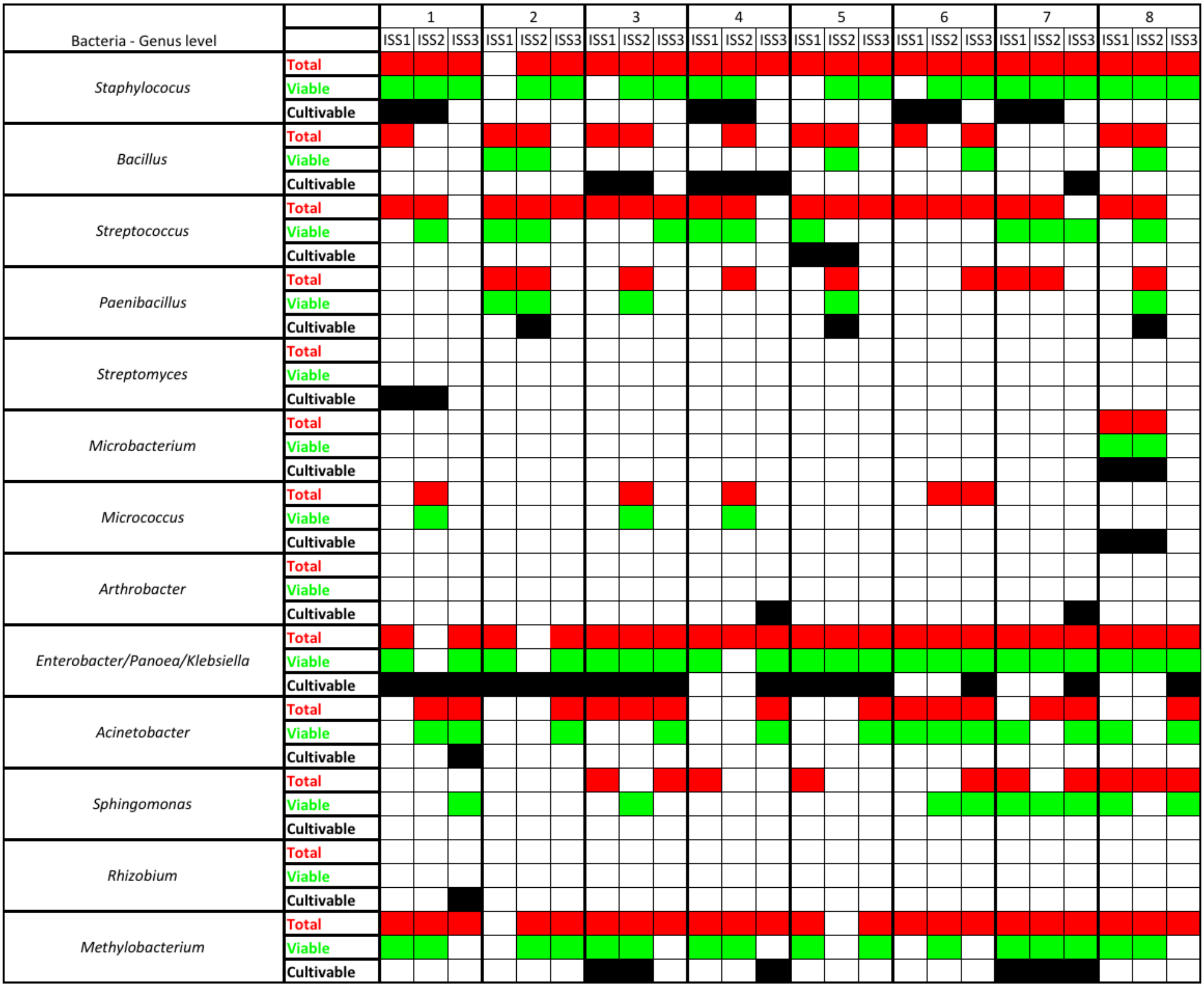
Bacteria - Genus level
1
2
3
4
5
6
7
8
ISS1 ISS2 ISS3
Total
Viable
Cultivable
Staphylococus
Bacillus
Streptococcus
Paenibacillus
Streptomyces
Microbacterium
Micrococcus
Arthrobacter
Enterobacter/Panoea/Klebsiella
Acinetobacter
Sphingomonas
Rhizobium
Methylobacterium

Fig. S11

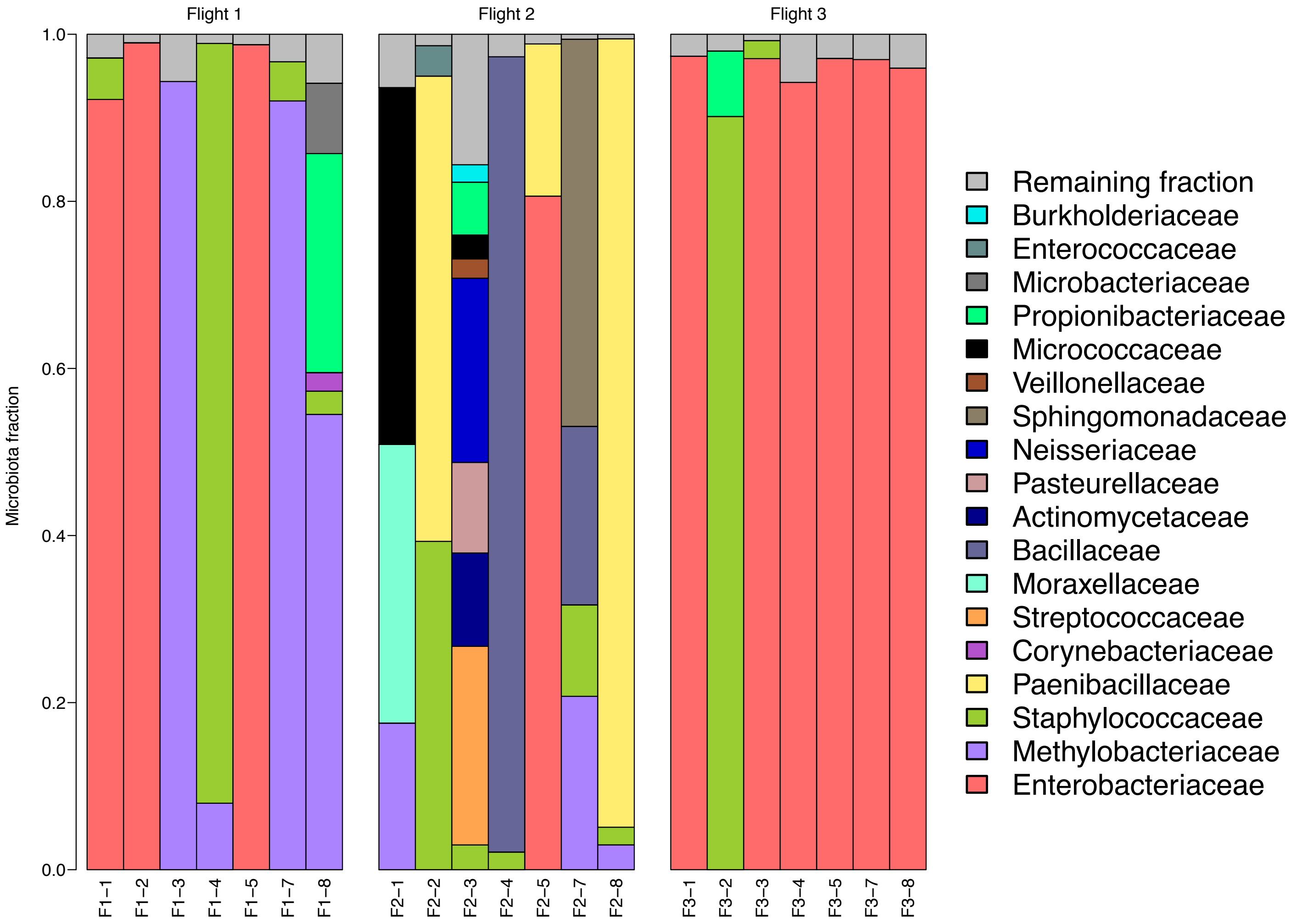
Flight 1
Flight 2
Flight 3
Microbiota fraction
1.0
0.8
0.6
0.4
0.2
0.0
F1−1
F1−2
F1−3
F1−4
F1−5
F1−7
F1−8
F2−1
F2−2
F2−3
F2−4
F2−5
F2−7
F2−8
F3−1
F3−2
F3−3
F3−4
F3−5
F3−7
F3−8
Remaining fraction
Burkholderiaceae
Enterococcaceae
Microbacteriaceae
Propionibacteriaceae
Micrococcaceae
Veillonellaceae
Sphingomonadaceae
Neisseriaceae
Pasteurellaceae
Actinomycetaceae
Bacillaceae
Moraxellaceae
Streptococcaceae
Corynebacteriaceae
Paenibacillaceae
Staphylococcaceae
Methylobacteriaceae
Enterobacteriaceae
