# Supplementary material for: Characterization of the total and viable bacterial and fungal communities associated with the International Space Station surfaces
Source: Microbiome. 2019 Apr 8;7:50. doi: 10.1186/s40168-019-0666-x (PMC6452512; doi:10.1186/s40168-019-0666-x)
Supplement: Supplementary file 3 — Table S1. Summary of results obtained from 16S rRNA gene amplicon sequencing. (DOCX 80 kb) [file 40168_2019_666_MOESM3_ESM.docx]

**Table S1**: Summary of results obtained from 16S rRNA gene amplicon sequencing of 24 wipes collected from 8 locations over 3 flight sampling sessions. Half of each processed wipe was treated with PMA in order to characterize intact/viable bacteria and the other half left untreated to characterize the total bacterial community (both intact/viable and dead/ruptured).

| Summary | Non- PMA treated | PMA treated |
| --- | --- | --- |
| Total reads | 5,434,563 | 4,900,415 |
| # ASVs | 515 | 403 |
| Phyla | (9)  Actinobacteria  Bacteroidetes  Deinococcus-Thermus  Firmicutes  Fusobacteria  Planctomycetes  Proteobacteria  Tenericutes  Unidentified | (7)  Actinobacteria  Bacteroidetes  Chloroflexi  Firmicutes  Planctomycetes  Proteobacteria  Unidentified |
| Top 5 Family (total counts) | *Enterobacteriaceae*  *Methylobacteriaceae*  *Staphylococcaceae*  *Paenibacillaceae*  *Corynebacteriaceae* | *Enterobacteriaceae*  *Methylobacteriaceae*  *Staphylococcaceae*  *Paenibacillaceae*  *Moraxellaceae* |
| Top 5 genera (total counts) | *Methylobacterium*  *Staphylococcus*  *Paenibacillus*  *Corynebacterium*  *Streptococcus* | *Methylobacterium*  *Staphylococcus*  *Paenibacillus*  *Acinetobacter*  *Streptococcus* |
| # of taxa summarized to the genus level | 109 | 76 |
