# Supplementary material for: Characterization of the total and viable bacterial and fungal communities associated with the International Space Station surfaces
Source: Microbiome. 2019 Apr 8;7:50. doi: 10.1186/s40168-019-0666-x (PMC6452512; doi:10.1186/s40168-019-0666-x)
Supplement: Supplementary file 4 — Table S4. List of unique sequences found on the ISS compared to Earth built environments. (DOCX 103 kb) [file 40168_2019_666_MOESM4_ESM.docx]

Additional File: Table S4:

Using the unrarefied data, we also compared the unique sOTUs present in the ISS data with the EMP and the built environment datasets to determine whether any sOTUs appear to be unique to the ISS. For this analysis, we were only able to examine ISS Flight 3 as it is highly sensitive to the primer. We removed any sOTUs observed in the ISS controls. There were 4 sOTUs that appear to be unique relative to the built environment, however they accounted for a very small total amount of the sequence mass (~0.0005% of the reads). The sOTUs are pasted below.

1. TACGGAGGATGCGAGCGTTATCCGGATTTATTGGGTTTAAAGGGAGCGCAGACGGTATGTTAAGTCAGCTGTGAAAGTTTGGGGCTCAAC
   1. Taxon identified: *Bacteroides* sp.
   2. <https://blast.ncbi.nlm.nih.gov/Blast.cgi?CMD=Get&RID=1VKMD4WU015>
   3. Number of samples the sequence was observed: 1
   4. Total number of observed reads: 2
2. TACGGAGGGCGAGAGCGTTACCCGGATTCACTGGGCGTAAAGGGCGTGTAGGCGGCCTGGGGCGTCCCATGTGAAAGACCACGGCTCAAC
   1. Taxon identified: *Thermus thermophilus*
   2. <https://blast.ncbi.nlm.nih.gov/Blast.cgi?CMD=Get&RID=1VKR38RU015>
   3. Number of samples the sequence was observed: 1
   4. Total number of observed reads: 33
3. TACGTAGGGGGCAAGCGTTGTCCGGAATTATTGGGCGTAAAGCGCGCGCAGGCGGTTCATTAAGTCTGGGGTTTAATCGCAGGGCTCAAC
   1. Taxon identified: *Paenibacillus thailandensis*
   2. <https://blast.ncbi.nlm.nih.gov/Blast.cgi?CMD=Get&RID=1VKT1ENH015>
   3. Number of samples the sequence was observed: 2
   4. Total number of observed reads: 7
4. TACGTAGGGGGCGAGCGTTATCCGGAATTATTGGGCGTAAAGGGTACGTAGGCGGCCAGATAAGTCAGGTGTGAAAGGCGTCGGCTCAAC
   1. Taxon identified: *Gottschalkia acidurici*
   2. <https://blast.ncbi.nlm.nih.gov/Blast.cgi?CMD=Get&RID=1VKVPDJ5015>
   3. Number of samples the sequence was observed: 1
   4. Total number of observed reads: 2

A single sOTU was unique in F3 compared to the EMP + the built environment. That is pasted below.

- TACGGAGGGCGAGAGCGTTACCCGGATTCACTGGGCGTAAAGGGCGTGTAGGCGGCCTGGGGCGTCCCATGTGAAAGACCACGGCTCAAC

1. Taxon identified: *Thermus thermophilus*
2. <https://blast.ncbi.nlm.nih.gov/Blast.cgi?CMD=Get&RID=1VKR38RU015>
   1. Number of samples the sequence was observed: 1
   2. Total number of observed reads: 33
