# Supplementary material for: Characterization of the total and viable bacterial and fungal communities associated with the International Space Station surfaces
Source: Microbiome. 2019 Apr 8;7:50. doi: 10.1186/s40168-019-0666-x (PMC6452512; doi:10.1186/s40168-019-0666-x)
Supplement: Supplementary file 5 — Table S2. Information pertaining to the efficiency of the different primers in detecting the most relatively abundant Family level taxa. (DOCX 18 kb) [file 40168_2019_666_MOESM5_ESM.docx]

**Table S2**: Information pertaining to the efficiency of the different primers in detecting the most relatively abundant Family level taxa.

^1^F3 is the primer used for Flight 3: 515fB and 806rB and F1/F2 was the primer used for Flight 1 and Flight 2: A519F and 802R

^2^Denotes the total number of sequences in the SILVA database that have been taxonomically assigned to that taxon.

^3^Denotes the number of sequences that match that taxon, with 0 mismatches, after amplification with the primer pair.

^4^The coverage was calculated with this formula: (# matches/total sequences found) * 100

This primer information was evaluated with the Testrprime system:

<https://www.arb-silva.de/search/testprime/>

| Organism | Primer  Pair^1^ | Total sequences found^2^ | # matches^3^ | Coverage (%)^4^ |
| --- | --- | --- | --- | --- |
| *Enterobacteriaceae* | F3  F1/F2 | 27966  27966 | 26039  26241 | 93.1  93.8 |
| *Methylobacteriaceae* | F3  F1/F2 | 1438  1438 | 1292  1308 | 89.8  91.0 |
| *Staphylococcaceae* | F3  F1/F2 | 7427  7427 | 5733  5841 | 77.2  78.6 |
| c_*Bacilli* | F3  F1/F2 | 48598  48598 | 41705  42434 | 85.8  87.3 |
| *Paenibacillaceae* | F3  F1/F2 | 3576  3576 | 3316  3357 | 92.7  93.9 |
| *Corynebacteriaceae* | F3  F1/F2 | 4833  4833 | 3759  3862 | 77.8  79.9 |
| *Streptococcaceae* | F3  F1/F2 | 8893  8893 | 7543  7678 | 84.8  86.3 |
| o_*Bacillales* | F3  F1/F2 | 30622  30622 | 26152  26685 | 85.3  87.0 |
| o_*Clostridiales* | F3  F1/F2 | 82141  82141 | 74476  74611 | 90.7  90.8 |
| *Moraxellaceae* | F3  F1/F2 | 11204  11204 | 10361  10425 | 92.5  93.0 |
| o_*Sphingomonadales* | F3  F1/F2 | 7711  3765 | 6981  7070 | 90.5  91.7 |
