# Supplementary material for: Characterization of the total and viable bacterial and fungal communities associated with the International Space Station surfaces
Source: Microbiome. 2019 Apr 8;7:50. doi: 10.1186/s40168-019-0666-x (PMC6452512; doi:10.1186/s40168-019-0666-x)
Supplement: Supplementary file 6 — Table S3. Environmental parameters for ISS samples. (DOCX 21 kb) [file 40168_2019_666_MOESM6_ESM.docx]

**Table S3:** Environmental parameters for ISS samples

| **Environmental parameters** | **Flight 1** | **Flight 2** | **Flight 3** | |
| --- | --- | --- | --- | --- |
| Sampling date | 3/4/15 | 5/15/15 | 5/5/16 | 5/6/16 |
| Scheduled Crew time | 255mins | 330mins | 140mins(S1) | 110mins(S2 andS3) |
| Ops (GMT/Time) | 0063/08.35-12.50 | 135/13.00-18.30 | 126/13.20-15.45 | 127/13.45-15.35 and 16.55-1845 |
| Vehicle (Ascent/Descent) | SpX-5/TMA-14A | SpX-6/SpX-6 | SpX-8/SpX-8 | |
| Crew | T. Virts | T. Virts | J. Willams | |
|  |  |  |  |  |
| **Radiation Measurements (total dose/day session)** |  |  |  |  |
| TEPC (SM P327) Dose Rate (uGy) | 268 | 380 | 162 | 311 |
| TEPC (SM P327) Dose Equivalent (uSv) | 590 | 828.4 | 520 | 736 |
| REM S/N 1009 (SSC 15-JPM) Dose Rate (uGy) | ND | 353 | 151 | 257 |
| REM S/N 1009 (SSC 15-JPM) Dose Equivalent (uSv) | ND | 776 | 517 | 515 |
| REM S/N 5001 (SSC 11-Col) Dose Rate (uGy) | ND | 305 | 148 | 217 |
| REM S/N 5001 (SSC 11-Col) Dose Eq (uSv) | ND | 449 | 292 | 391 |
| REM S/N 1007 (SSC 9-US Lab) Dose Rate (uGy) | 192 | 235 | 152 | 208 |
| REM S/N 1007 (SSC 9-US Lab) Dose Eq (uSv) | 360 | 462 | 346 | 430 |
| REM S/N 1005 (SSC 7- JPM) Dose Rate (uGy) | ND | 279 | ND | ND |
| REM S/N 1005 (SSC 7-JPM) Dose Eq (uSv) | ND | 584 | ND | ND |
| REM S/N 2001 (SSC 6-Cupola) Dose Rate (uGy) | 248 | 301 | ND | ND |
| REM S/N 2001 (SSC 6-Cupola) Dose Eq (uSv) | 413 | 593 | ND | ND |
| REM S/N 1003 (Node 1) Dose Rate (uGy) | 283 | ND | ND | ND |
| REM S/N 1003 (Node 1) Dose Eq (uSv) | 518 | ND | ND | ND |
|  |  |  |  |  |
|  |  |  |  |  |
| ND: Unit was not working at the time of the sampling session |  |  |  |  |
|  |  |  |  |  |
| **Typical Terrestrial and Spaceflight-related radiation exposures (uSv)** |  |  |  |  |
| Exposure from typical chest X-ray (per exposure) | 100 | 100 | 100 | 100 |
| Exposure during a typical trans-Altlantic airline flight | 120 | 120 | 120 | 120 |
| Skin Dose onboard the ISS during Solar Maximum (per day) | 500 | 500 | 500 | 500 |
| Skin Dose onboard the ISS during Solar Minimum (per day) | 1000 | 1000 | 1000 | 1000 |
| Exposure from living in Houston, TX (per 6 months) | 500 | 500 | 500 | 500 |
| Exposure from living in Denver, CO (per 6 months) | 1000 | 1000 | 1000 | 1000 |
| Exposure limit for U.S. general public (per 6 months) | 2500 | 2500 | 2500 | 2500 |
|  |  |  |  |  |
|  |  |  |  |  |
| **Temperature of ISS Modules (Deg C)** |  |  |  |  |
| Zvezda (Service Module) Cabin Temp | 26 | 24.7 | 27.2 | 26.4 |
| Harmony (Node 2) CCAA | 22.6 | 22.8 | 20.8 | 20.7 |
| Tranquility (Node 3) CCAA | 22.9 | 22.8 | 21.9 | 21.8 |
| Destiny (US Laboratory) CCAA | 22.2 | 21.8 | 21.4 | 21.1 |
| Columbus (EU Laboratory) Cabin Temp | 23 | 23 | 22 | 19.8 |
| ISS Total Pressure (kPa)* | 101.008 | 101.56 |  |  |
| Humidity (%)** | 40.2 | 39.25 |  |  |
| Partial Pressure (pp) of Air constituents (kPa) |  |  |  |  |
| Zvezda (Service Module) ppO_2_ | 21.581 | 20.546 | 20.822 | 20.546 |
| Zvezda (Service Module) ppCO_2_ | 0.3723 | 0.2689 | 0.4132 | 0.5066 |
| Zvezda (Service Module) ppH_2_O | 1.0342 | 0.8963 | 1.0342 | 1.0342 |
| Columbus (EU Laboratory) ppO_2_ | 21.47 | 20.733 | 19.665 | 19.332 |
| Columbus (EU Laboratory) ppCO_2_ | 0.4275 | 0.3516 | 0.3999 | 0.3786 |
| JEM: Japanese Experimental Module |  |  |  |  |
| *US Lab, Columbus, JEM Total Pressure |  |  |  |  |
| **Columbus |  |  |  |  |
| CCAA=Common Cabin Air Assembly |  |  |  |  |
